# Luminescent *cis-*Bis(bipyridyl)ruthenium(II) Complexes with 1,2-Azolylamidino Ligands: Photophysical, Electrochemical Studies, and Photocatalytic Oxidation of Thioethers

**DOI:** 10.1021/acs.inorgchem.0c03389

**Published:** 2021-04-27

**Authors:** Elena Cuéllar, Alberto Diez-Varga, Tomás Torroba, Pablo Domingo-Legarda, José Alemán, Silvia Cabrera, Jose M. Martín-Alvarez, Daniel Miguel, Fernando Villafañe

**Affiliations:** † GIR MIOMeT-IU Cinquima-Química Inorgánica, Facultad de Ciencias, Campus Miguel Delibes, Universidad de Valladolid, 47011 Valladolid, Spain; ‡ Departamento de Química, Facultad de Ciencias, Universidad de Burgos, 09001 Burgos, Spain; § Departamento de Química Inorgánica, Facultad de Ciencias, Universidad Autónoma de Madrid, 28049 Madrid, Spain

## Abstract

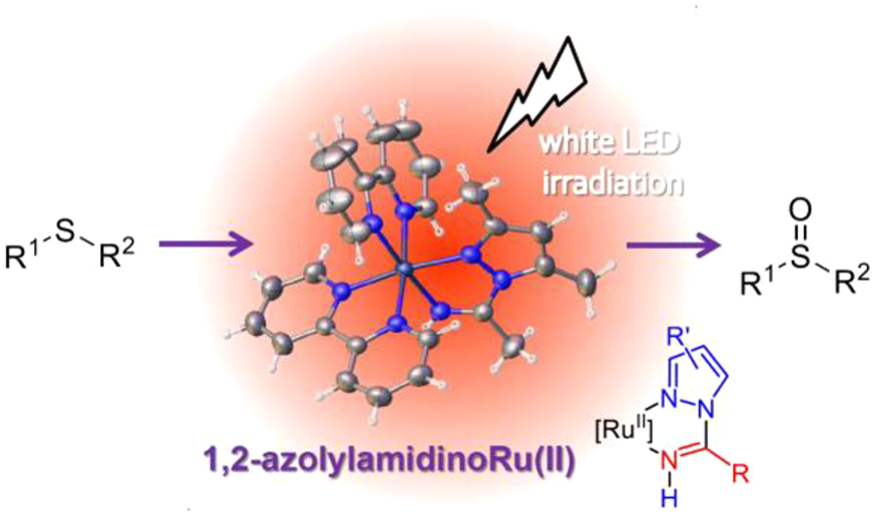

New 1,2-azolylamidino complexes *cis*-[Ru(bipy)_2_(NH=C(R)az*-κ^2^*N*,*N*)](OTf)_2_ (R = Me, Ph; az* = pz, indz, dmpz) are synthesized via chloride abstraction after a subsequent base-catalyzed coupling of a nitrile with the previously coordinated 1,2-azole. The synthetic procedure allows the easy obtainment of complexes having different electronic and steric 1,2-azoylamidino ligands. All of the compounds have been characterized by ^1^H, ^13^C, and ^15^N NMR and IR spectroscopy and by monocrystal X-ray diffraction. Photophysical studies support their phosphorescence, whereas their electrochemistry reveals reversible Ru^II^/Ru^III^ oxidations between +1.13 and +1.25 V (vs SCE). The complexes have been successfully used as catalysts in the photooxidation of different thioethers, the complex *cis*-[Ru(bipy)_2_(NH=C(Me)dmpz-κ^2^*N*,*N*)]^2+^ showing better catalytic performance in comparison to that of [Ru(bipy)_3_]^2+^. Moreover, the significant catalytic performance of the dimethylpyrazolylamidino complex is applied to the preparation of the drug modafinil, which is obtained using ambient oxygen as an oxidant. Finally, mechanistic assays suggest that the oxidation reaction follows a photoredox route via oxygen radical anion formation.

## Abstract 2

Complexes with the 1,2-azolylamidino ligand coordinated to the *cis*-Ru^II^(bipy)_2_ fragment are phosphorescent, their redox properties display reversible Ru^II^/Ru^III^ oxidations, and they are catalysts in the photooxidation of different thioethers. [Ru(bipy)_2_(NH=C(Me)dmpz-κ^2^*N*,*N*)]^2+^ presents a better catalytic performance in comparison to that of [Ru(bipy)_3_]^2+^. The 1,2-azolylamidino complexes may be readily synthesized, and their electrochemical, luminescent, and catalytic activity may be adequately tuned by varying the steric and electronic effects of the 1,2-azole and nitrile precursors.

## Introduction

During the last few decades ruthenium complexes with polypyridyl ligands have been luminescent metal complexes studied in great detail. Interest in ruthenium chemistry awoke in the late 1950s and 1960s with two publications: the first report on the luminescent properties of the complex [Ru(bipy)_3_]^2+^ (bipy = 2,2′- bipyridyl) by Paris and Brandt^1^ and that of the mixed-valence dinuclear complex [(NH_3_)_5_Ru(μ-pyrazine)Ru(NH_3_)_5_]^5+^ by Creutz and Taube.^2^ The interest in ruthenium complexes with polypyridyl ligands extended broadly during the mid- to late-1970s, after the publication of the dissociation of water into hydrogen and oxygen facilitated by the association of the excited state and the electrochemical behavior of the compound [Ru(bipy)_3_]^2+^.^3^ The initial expectations of this result soon faded, but these promising physicochemical properties encouraged research in other areas of interest,^4^ such as their photocatalytic activity toward the reduction of CO_2_^5^ or their function as molecular switches^6^ or cation sensors.^7^ The application of these Ru(II) complexes as photoprobes or photochemical reagents for biomolecules was soon also found.^8−16^ This is related to the discovery of cisplatin and its antitumor effect, which led to the rapid spread of new therapeutic agents based on metals different from platinum.^17^

Another interesting aspect of these Ru(II) complexes is their use as visible-light photocatalysts, especially in the activation of organic molecules.^18−22^ Some crucial aspects are, first of all, these complexes absorb visible light to give a stable and long-lived photoexcited state,^23,24^ second, the lifetime of the excited species is adequately long to take part in electron-transfer reactions competing with deactivation processes,^25^ and finally, their excitation states are very powerful single-electron-transfer reagents.^18^ One of the many reactions catalyzed by these types of complexes is the oxidation of sulfides in order to synthesize sulfoxides.^26−33^ The latter have a plethora of applications as chiral auxiliaries in asymmetric synthesis^34^ and are located in many drugs as well as in natural products.^35^ From an industrial point of view, the oxidation of sulfides is performed with complexes as catalysts and with peroxides or peracids as oxidants. Nevertheless, this approach presents two main drawbacks: the production of sulfones as byproducts due to overoxidation processes and the difficulties in handling peroxides, which are explosive reagents. In contrast, the oxidation of sulfides, via photocatalysis, using atmospheric O_2_ represents a more secure option.

**Scheme 1:**
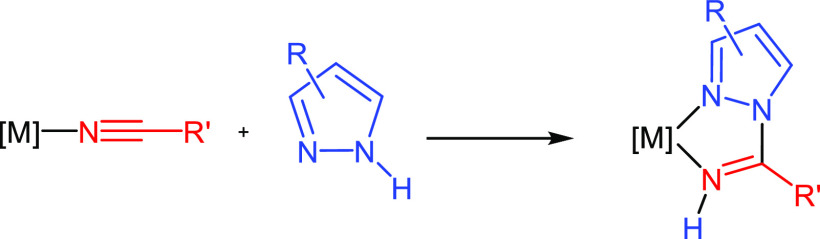
Coupling Reaction between a Coordinated Nitrile and a 1,2-Azole to Form a 1,2-Azolylamidino Ligand

Currently, the search for appropriate *N*-aromatic donor chelate ligands in order to obtain the desired chemical or physical properties of the Ru(II) polypyridyl complexes is one of the main topics of this field. 1,2-Azolylamidino ligands (Scheme 1) have been shown to be an important family of ligands, due to not only their electronic delocalization but also the different features of the two nitrogen donor atoms. However, as far as we know, the coordination of such ligands to Ru(II) polypyridyl systems has not been previously reported. The only precedents of bidentate ligands with 1,2-azole moieties also contained 2-pyridyl fragments and were reported several decades ago.^36,37^ The introduction of substituents in the diimine ligands more commonly employed usually requires difficult and often tiresome synthetic methods. The advantage of 1,2-azolylamidino ligands is evident in this aspect: their *in situ* synthesis (Scheme 1) enables the easy production of new bidentate chelates with different electronic and steric properties only by choosing the appropriate nitrile and 1,2-azole, both being readily available. Our previous study on the mechanism describing the coupling of 1,2-azoles and nitriles mediated by complexes broadened the range of synthetic possibilities.^38^ The acidic hydrogen in the 1,2-azolylamidino ligand is also interesting, since the NH moiety may cause further reactivity. In this regard, the participation of this amino group in intra- or intermolecular noncovalent interactions might lead to the stabilization of a concrete isomer in the first case or to fascinating supramolecular assemblies in the latter case.

Herein we describe the synthesis of new Ru(II) complexes obtained by coordination of the 1,2-azolylamidino ligand to the *cis*-bis(bipyridyl)ruthenium(II) moiety, as well as the spectroscopic, electrochemical, and photophysical behavior of the complexes obtained. The application of the compounds as photocatalysts using O_2_ is also evaluated in the oxidation of thioethers.

## Experimental Section

### General Remarks

**Figure 1:**
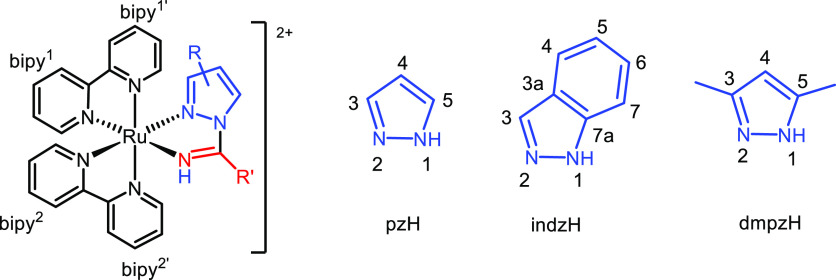
Atomic numbering of bipy, pzH, indzH, and dmpzH for NMR assignment.

All manipulations were carried out under N_2_ following conventional Schlenk procedures. Solvents were purified according to standard methods. Complexes **1** and **2** were obtained as previously described by us.^39^ The rest of the reagents were purchased from the usual commercial suppliers and used as received. Infrared spectra were recorded with a Bruker Tensor 27 FTIR instrument. The abbreviations used to indicate intensity are w = weak, m = medium, s = strong, and vs = very strong. NMR spectra were recorded on a 500 MHz Agilent DD2 or 400 MHz Agilent MR apparatus in the Laboratorio de Técnicas Instrumentales (LTI, Universidad de Valladolid), using (CD_3_)_2_CO as the solvent at room temperature (rt) unless otherwise indicated. ^1^H, ^13^C, and ^15^N NMR chemical shifts (δ) are reported in parts per million (ppm) using the residual solvent peak as an internal reference and are referenced to tetramethylsilane (TMS, for ^1^H and ^13^C NMR) or to nitromethane (CH_3_NO_2_, for ^15^N NMR). Coupling constants (*J*) are reported in Hz. Abbreviations used to indicate multiplicity are s = singlet, d = doublet, ddd = doublet of doublets of doublets, dt = doublet of triplets, t = triplet, and m = multiplet. The complete assignment (Figure 1) of the ^1^H NMR spectra was supported by COSY and TOCSY and NOESY homonuclear ^1^H–^1^H correlations, whereas the assignment of ^13^C{^1^H} and ^15^N NMR data was supported by HMBC and HSQC heteronuclear experiments. Elemental analyses were performed on a Thermo Fisher Scientific EA Flash 2000 instrument.

### *cis*-[Ru(bipy)_2_(NH=C(Me)pz-κ^2^N,N)](OTf)_2_ (**3a**)

AgOTf (0.026 g, 0.1 mmol) was added to a solution of **1a** (0.068 g, 0.1 mmol) in MeCN (5 mL). A 100 μL portion of aqueous 0.02 M NaOH (0.002 mmol) was then added, and the mixture was stirred at rt for 24 h in the absence of light. The reaction mixture was filtered to remove solid AgCl and dried *in vacuo*. The red residue was crystallized in MeCN/Et_2_O at −20 °C, giving a red microcrystalline solid, which was decanted, washed with Et_2_O (3 × 3 mL approximately), and dried *in vacuo*: yield 0.059 g (72%). In order to obtain a monocrystalline solid, a slight stoichiometric excess of NH_4_PF_6_ was added to a solution of the complex in acetone, giving [Ru(bipy)_2_(NH=C(Me)pz-κ^2^*N*,*N*)](PF_6_)_2_ as a red monocrystalline solid. ^1^H NMR (500 MHz, acetone-*d*_6_): δ 11.37 (s, NH, 1 H), 8.90–8.84 (m, H^6′^ bipy^1^ and H^5^ pz, 2 H), 8.81–8.74 (m, H^3′^ bipy^1^, H^3^ bipy^2^ and H^3′^ bipy^2^, 3 H), 8.70 (d, *J* = 7.8 Hz, H^3^ bipy^1^, 1 H), 8.29–8.18 (m, H^4′^ bipy^2^ and H^4′^ bipy^1^, 2 H), 8.14 (ddd, *J* = 8.2, 7.6, 1.5 Hz, H^4^ bipy^2^, 1 H), 8.12–8.04 (m, H^6′^ bipy^2^, and H^4^ bipy^1^, 1 H), 8.01 (ddd, *J* = 5.7, 1.5, 0.8 Hz, H^6^ bipy^2^, 1 H), 7.93 (ddd, *J* = 5.7, 1.4, 0.8 Hz, H^6^ bipy^1^, 1 H), 7.74–7.65 (m, H^5′^ bipy^1^, H^3^ pz, and H^5′^ bipy^2^, 3 H), 7.51 (ddd, *J* = 7.5, 5.6, 1.3 Hz, H^5^ bipy^2^, 1 H), 7.45 (ddd, *J* = 7.5, 5.6, 1.3 Hz, H^5^ bipy^1^, 1 H), 6.86 (dd, *J* = 3.2, 2.1 Hz, H^4^ pz, 1 H), 3.07 (d, *J* = 1.1 Hz, NH=C*CH*_*3*_, 3 H). ^13^C NMR (126 MHz, acetone): δ 163.05 (1C, NH=*C*CH_3_), 157.95 (1C, C^2^ bipy^1^), 157.63 (1C, C^2^ bipy^2^), 157.46 (2C, C^2′^ bipy^1^ and C^2′^ bipy^2^), 153.53 (1C, C^6′^ bipy^1^), 152.21 (1C, C^6^ bipy^1^), 151.87 (1C, C^6^ bipy^2^), 151.69 (1C, C^6′^ bipy^2^), 145.45 (1C, C^3^ pz), 137.70 (1C, C^4′^ bipy^2^), 137.57 (1C, C^4′^ bipy^1^), 137.42 (1C, C^4^ bipy^2^), 137.32 (1C, C^4^ bipy^1^), 134.06 (1C, C^5^ pz), 127.68 (1C, C^5′^ bipy^2^), 127.50 (1C, C^5′^ bipy^1^), 127.07 (1C, C^5^ bipy^2^), 126.84 (1C, C^5^ bipy^1^), 123.92 (1C, C^3′^ bipy^1^), 123.89 (1C, C^3^ bipy^2^), 123.75 (1C, C^3′^ bipy^2^), 123.70 (1C, C^3′^ bipy^1^), 111.53 (1C, C^4^ pz), 17.94 (1C, NH=C*CH*_*3*_). IR (solid, cm^–1^): 3237 m, 3117 w, 3092 w, 2929 w, 2290 w, 2164 w, 2140 w, 2051 w, 1981 w, 1913 w, 1695 m, 1638 m, 1605 w, 1568 w, 1523 w, 1466 s, 1445 s, 1420 s, 1402 m, 1378 w, 1329 w, 1315 w, 1263 vs, 1222 vs, 1141 vs, 1071 m, 1048 m, 1026 vs, 958 s, 911 m, 850 m, 801 w, 762 vs, 744 s, 429 vs, 681 w, 659 w, 632 vs. Anal. Calcd for C_27_H_23_F_6_N_7_O_6_RuS_2_: C, 39.51; H, 2.82; N, 11.95; S, 7.81. Found: C, 39.85; H, 2.74; N, 11.84; S, 8.19.

### *cis*-[Ru(bipy)_2_(NH=C(Me)indz-κ^2^*N*,*N*)](OTf)_2_ (**3b**)

A procedure similar to that for **3a**, using **1b** (0.072 g, 0.1 mmol) as the starting material, but without addition of the NaOH solution, gave 0.065 g (75%) of **3b** as a red microcrystalline solid. ^1^H NMR (500 MHz, acetone-*d*_6_): δ 10.99 (s, NH, 1 H), 8.90 (d, *J* = 5.6 Hz, H^6′^ bipy^1^, 1 H), 8.80 (d, *J* = 8.2 Hz, H^3′^ bipy^1^, H^3^ bipy^1^ and H^3′^ bipy^2^, 3 H), 8.73 (d, *J* = 8.2 Hz, H^3^ bipy^2^, 1 H), 8.37 (d, *J* = 0.8 Hz, H^3^ indz, 1 H), 8.28–8.21 (m, H^6′^ and H^4′^ bipy^2^, 2 H), 8.23–8.14 (m, H^4′^ bipy^1^, H^4^ bipy^1^, and H^7^ indz, 3 H), 8.10 (t, *J* = 7.9 Hz, H^4^ bipy^2^, 1 H), 8.06 (d, *J* = 4.9 Hz, H^6^ bipy^1^, 1 H), 7.95 (d, *J* = 6.4 Hz, H^6^ bipy^2^, 1 H), 7.84 (d, *J* = 8.1 Hz, H^4^ indz, 1 H), 7.71 (ddd, *J* = 8.5, 7.2, 1.1 Hz, H^6^ indz, 1 H), 7.66 (ddd, *J* = 7.0, 5.6, 1.3 Hz, H^5′^ bipy^1^, 1 H), 7.60 (ddd, *J* = 7.0, 5.6, 1.3 Hz, H^5′^ bipy^2^, 1 H), 7.55 (ddd, *J* = 7.0, 5.6, 1.3 Hz, H^5^ bipy^1^, 1 H), 7.50–7.42 (m, H^5^ bipy^2^ and H^5^ indz, 2 H), 3.37 (s, NH=C*CH*_*3*_, 3 H). ^13^C NMR (126 MHz, acetone-*d*_6_): δ 163.68 (1C, NH=*C*CH_3_), 157.57 (2C, C^2^ bipy^2^ and C^2^ bipy^1^), 157.39 (1C, C^2′^ bipy^1^), 157.29 (1C, C^2′^ bipy^2^), 153.53 (1C, C^6′^ bipy^1^), 151.95 (1C, C^6^ bipy^2^), 151.82 (1C, C^6^ bipy^1^), 151.79 (1C, C^6′^ bipy^2^), 142.87 (1C, C^3^ indz), 140.03 (1C, C^7a^ indz), 137.73 (1C, C^4′^ bipy^2^), 137.70 (1C, C^4′^ bipy^1^), 137.60 (1C, C^4^ bipy^1^), 137.46 (1C, C^4^ bipy^2^), 130.09 (1C, C^6^ indz), 127.60 (1C, C^5′^ bipy^2^), 127.49 (1C, C^5′^ bipy^1^), 127.20 (1C, C^5^ bipy^1^), 126.87 (1C, C^5^ bipy^2^), 126.64 (1C, C^3a^ indz), 124.72 (1C, C^5^ indz), 124.04 (2C, C^3^ bipy^1^ and C^3′^ bipy^1^), 123.79 (1C, C^3′^ bipy^2^), 123.73 (1C, C^3^ bipy^2^), 121.69 (1C, C^4^ indz), 112.16 (1C, C^7^ indz), 20.44 (1C, NH=C*CH*_*3*_). ^15^N NMR (51 MHz, acetone-*d*_6_): δ −119.46 (1N, N^2^ indz), −129.11 (1N, N^1′^ bipy^1^), −129.88 (1N, N^1′^ bipy^2^), −130.40 (1N, N^1^ bipy^1^), −133.12 (1N, N^1^ bipy^2^), −166.05 (1N, N^1^ indz), −185.22 (1N, NH). IR (solid, cm^–1^): 3190 m, 3090 w, 2989 w, 2935 w, 2626 w, 2288 w, 2187 w, 2164 w, 2140 w, 2113 w, 2051 w, 2018 w, 1981 w, 1903 w, 1853 w, 1781 w, 1694 s, 1632 m, 1602 w, 1509 w, 1462 s, 1435 s, 1420 s, 1348 w, 1251 vs, 1224 vs, 1191 s, 1141 vs, 1081 m, 1029 vs, 913 w, 889 m, 858 m, 836 m, 805 w, 770 vs, 749 vs, 730 s, 714 m, 661 w, 632 vs. Anal. Calcd for C_31_H_25_F_6_N_7_O_6_RuS_2_: C, 42.76; H, 2.89; N, 11.26; S, 7.36. Found: C, 43.01; H, 2.83; N, 11.44; S, 7.80.

### *cis*-[Ru(bipy)_2_(NH=C(Me)dmpz-κ^2^N,N)](OTf)_2_ (**3c**)

The same procedure as for **3a**, using **1c** (0.069 g, 0.1 mmol) as the starting material, gave 0.060 g (71%) of **3c** as a red microcrystalline solid. ^1^H NMR (500 MHz, acetone-*d*_6_): δ 11.13 (s, NH, 1 H), 8.85 (d, *J* = 4.9 Hz, H^6′^ bipy^1^, 1 H), 8.82–8.74 (m, H^3^ bipy^1^,H^3′^ bipy^1^ and H^3′^ bipy^2^, 3 H), 8.67 (dt, *J* = 8.2, 1.0 Hz, H^3^ bipy^2^, 1 H), 8.29–8.19 (m, H^4′^ bipy^1^ and H^4′^ and H^6′^ bipy^2^, 3 H), 8.13 (td, *J* = 8.2, 1.5 Hz, H^4^ bipy^1^, 1 H), 8.09–8.01 (m, H^4^ bipy^2^ and H^6^ bipy^1^, 2 H), 7.78 (d, *J* = 4.3 Hz, H^6^ bipy^2^, 1 H), 7.73 (ddd, *J* = 7.6, 5.6, 1.3 Hz, H^5′^ bipy^1^, 1 H), 7.69 (ddd, *J* = 7.1, 5.6, 1.3 Hz, H^5′^ bipy^2^, 1 H), 7.51 (ddd, *J* = 7.2, 5.7, 1.3 Hz, H^5^ bipy^1^, 1 H), 7.41 (ddd, *J* = 7.0, 5.7, 1.3 Hz, H^5^ bipy^2^, 1 H), 6.44 (s, H^4^ dmpz, 1 H), 3.11 (s, NH=C*CH*_*3*_, 3 H), 2.81 (s, CH_3_^5^ dmpz, 3 H), 1.64 (s, CH_3_^3^ dmpz, 3 H). ^13^C NMR (126 MHz, acetone): δ 164.45 (1C, NH=*C*CH_3_), 157.97 (1C, C^2^ bipy^2^), 157.53 (1C, C^2^ bipy^1^), 157.42 (1C, C^2′^ bipy^2^), 157.31 (1C, C^2′^ bipy^1^), 157.13 (1C, C^3^ dmpz), 153.01 (1C, C^6′^ bipy^1^), 152.18 (1C, C^6′^ bipy^2^), 151.97 (1C, C^6^ bipy^2^), 151.65 (1C, C^6^ bipy^1^), 146.75 (1C, C^5^ dmpz), 137.46 (2C, C^4′^ bipy^1^ and C^4′^ bipy^2^), 137.32 (2C, C^4^ bipy^1^ and C^4^ bipy^2^), 127.73 (1C, C^5′^ bipy^2^), 127.52 (1C, C^5′^ bipy^1^), 127.39 (1C, C^5^ bipy^1^), 126.90 (1C, C^5^ bipy^2^), 124.19 (1C, C^3^ bipy^1^), 123.98 (1C, C^3′^ bipy^1^), 123.77 (1C, C^3′^ bipy^2^), 123.66 (1C, C^3^ bipy^2^), 113.58 (1C, C^4^ dmpz), 20.82 (1C, NH=C*CH*_*3*_), 13.56 (1C, CH_3_^5^ dmpz), 11.49 (1C, CH_3_^3^ dmpz). ^15^N NMR (51 MHz, acetone-*d*_6_): δ −126.43 (1N, N^1^ bipy^1^), −127.55 (1N, N^1′^ bipy^1^), −129.02 (1N, N^1′^ bipy^2^), −133.32 (1N, N^1^ bipy^2^), −141.76 (1N, N^2^ dmpz), −151.25 (1N, N^1^ dmpz), −177.07 (1N, NH). IR (solid, cm^–1^): 3229 m, 3114 m, 3086 m, 2994 w, 2637 w, 2288 w, 2164 w, 2146 w, 2112 w, 2050 w, 1981 w, 1921 w, 1732 m, 1633 m, 1606 m, 1573 m, 1468 s, 1446 s, 1415 vs, 1387 w, 1361 m, 1316 w, 1252 vs, 1221 vs, 1151 vs, 1101 m, 1073 w, 1050 m, 1028 vs, 993 m, 962 m, 891 w, 833 w, 809 w, 766 m, 756 vs, 732 s, 684 w, 661 m, 635 vs. Anal. Calcd for C_29_H_27_F_6_N_7_O_6_RuS_2_: C, 41.04; H, 3.21; N, 11.55; S, 7.55. Found: C, 41.26; H, 3.15; N, 11.87; S, 8.15.

### *cis*-[Ru(bipy)_2_(NH=C(Ph)pz-κ^2^*N*,*N*)](OTf)_2_ (**4a**)

PhCN (100 μL) was added to a solution of **2a** (0.080 g, 0.1 mmol) in Me_2_CO (5 mL). A 100 μL portion of an aqueous 0.02 M solution of NaOH (0.002 mmol) was then added, and the mixture was stirred at rt for 24 h. The red solution was crystallized in acetone/Et_2_O at −20 °C, giving a red microcrystalline solid, which was decanted, washed with Et_2_O (3 × 3 mL approximately), and dried *in vacuo*: yield 0.059 g (67%). ^1^H NMR (400 MHz, acetone-*d*_6_): δ 11.77 (s, NH, 1 H), 8.95 (d, *J* = 5.3 Hz, H^6′^ bipy^1^, 1 H), 8.84–8.76 (m, H^3′^ bipy^1^ and H^3′^ bipy^2^, 2 H), 8.72 (m, H^3^ bipy^1^ and H^3^ bipy^2^, 2 H), 8.65 (d, *J* = 3.4 Hz, H^5^ pz, 1 H), 8.29–8.21 (m, H^6′^ bipy^2^, H^4′^ bipy^1^ and H^4′^ bipy^2^, 3 H), 8.16 (td, *J* = 7.8, 1.5 Hz, 1 H), 8.14–8.10 (m, H^4^ bipy^1^, 1 H), 8.09–8.03 (m, H^6^ bipy^2^, 1 H), 7.97 (dt, *J* = 7.2, 1.3 Hz, H^6^ bipy^1^ and *o*-C_6_H_5_, 3 H), 7.80 (d, *J* = 2.1 Hz, H^3^ pz, 1 H), 7.79–7.69 (m, H^5′^ bipy^1^, H^5′^ bipy^2^ and *p*-C_6_H_5_, 3 H), 7.63 (ddd, *J* = 8.8, 6.8, 1.5 Hz, *m*-C_6_H_5_, 2 H), 7.53 (ddt, *J* = 8.4, 6.9, 2.1 Hz, H^5^ bipy^1^, 1 H), 7.46 (ddd, *J* = 7.6, 4.4, 1.5 Hz, H^5^ bipy^2^, 1 H), 6.89 (dd, *J* = 3.4, 2.0 Hz, H^4^ pz, 1 H). ^13^C NMR (101 MHz, acetone): δ 163.85 (1C, NH=*C*Ph), 157.99 (1C, C^2^ bipy^1^), 157.55 (1C, C^2^ bipy^2^), 157.44 (2C, C^2′^ bipy^1^ and C^2′^ bipy^2^), 153.45 (1C, C^6′^ bipy^1^), 152.31 (1C, C^6^ bipy^1^), 151.90 (1C, C^6^ bipy^2^), 151.72 (1C, C^6′^ bipy^2^), 146.03 (1C, C^3^ pz), 137.95 (1C, C^4′^ bipy^2^), 137.82 (1C, C^4′^ bipy^1^), 137.64 (1C, C^4^ bipy^2^), 137.47 (1C, C^4^ bipy^1^), 135.41 (1C, C^5^ pz), 132.94 (1C, *p*-C_6_H_5_), 129.38 (2C, *m*-C_6_H_5_), 129.23 (1C, *ipso*-C_6_H_5_), 128.92 (2C, *o*-C_6_H_5_), 127.88 (1C, C^5′^ bipy^2^), 127.69 (1C, C^5′^ bipy^1^), 127.17 (1C, C^5^ bipy^2^), 126.85 (1C, C^5^ bipy^1^), 124.08 (1C, C^3′^ bipy^1^), 124.00 (1C, C^3′^ bipy^2^), 123.81 (1C, C^3^ bipy^2^), 123.71 (1C, C^3^ bipy^1^), 112.05 (1C, C^4^ pz). ^15^N NMR (51 MHz, acetone-*d*_6_): δ −128.17 (1N, N^1′^ bipy^1^), −131.19 (1N, N^1′^ bipy^2^), −131.53 (1N, N^1^ bipy^2^), −134.46 (1N, N^1^ bipy^1^), −141.51 (1N, N^1^ pz), −147.69 (1N, N^2^ pz), −169.98 (1N, NH). IR (solid, cm^–1^): 3501 w, 3114 m, 3082 m, 2977 w, 2870 w, 2644 w, 2324 w, 2288 w, 2187 w, 2164 w, 2149 w, 2112 w, 2051 w, 2011 w, 1981 w, 1916 w, 1607 m, 1571 w, 1521 w, 1495 w, 1467 m, 1448 s, 1433 s, 1387 m, 1315 w, 1255 vs, 1222 vs, 1147 vs, 1089 s, 1027 vs, 980 m, 895 m, 849 w, 804 w, 763 vs, 731 s, 705 s, 662 w, 634 vs. Anal. Calcd for C_32_H_25_F_6_N_7_O_6_RuS_2_: C, 43.53; H, 2.86; N, 11.11; S, 7.27. Found: C, 43.73; H, 3.00; N, 10.89; S, 7.11.

### *cis*-[Ru(bipy)_2_(NH=C(Ph)indz-κ^2^*N*,*N*)](OTf)_2_ (**4b**)

A procedure similar to that for **4a**, using **2b** (0.085 g, 0.1 mmol) as the starting material, but without addition of the NaOH solution, gave 0.067 g (72%) of **4b** as a red microcrystalline solid. ^1^H NMR (500 MHz, acetone-*d*_6_, 278 K): δ 11.26 (s, NH, 1 H), 9.04 (d, *J* = 6.3 Hz, H^6′^ bipy^1^, 1 H), 8.85 (d, *J* = 8.2 Hz, H^3^ bipy^1^ and H^3′^ bipy^1^, 2 H), 8.82 (d, *J* = 8.2 Hz, H^3′^ bipy^2^, 1 H), 8.75 (d, *J* = 8.1 Hz, H^3^ bipy^2^, 1 H), 8.57 (d, *J* = 5.6 Hz, H^6′^ bipy^2^, 1 H), 8.49 (s, H^3^ indz, 1 H), 8.29 (td, *J* = 8.0, 1.5 Hz, H^4′^ bipy^2^, 1 H), 8.26–8.19 (m, H^4^ bipy^1^ and H^4′^ bipy^1^, 2 H), 8.17–8.09 (m, H^6^ bipy^1^, H^4^ bipy^2^ and *o*-C_6_H_5_, 3 H), 8.02 (d, *J* = 6.4 Hz, H^6^ bipy^2^, 1 H), 7.85–7.63 (m, H^4^ indz, H^5′^ bipy^1^, H^5′^ bipy^2^, *p*-C_6_H_5_, *m*-C_6_H_5_ and *o*-C_6_H_5_, 7 H), 7.60 (ddd, *J* = 7.5, 5.6, 1.3 Hz, H^5^ bipy^1^, 1 H), 7.50 (ddd, *J* = 7.3, 5.7, 1.3 Hz, H^5^ bipy^2^, 1 H), 7.45–7.39 (m, H^5^ indz and H^6^ indz, 2 H), 6.66 (s, H^7^ indz, 1 H). ^1^H NMR (500 MHz, acetone-*d*_6_, 243 K): δ 11.26 (s, NH, 1 H), 9.04 (d, *J* = 6.3 Hz, H^6′^ bipy^1^, 1 H), 8.85 (d, *J* = 8.2 Hz, H^3^ bipy^1^ and H^3′^ bipy^1^, 2 H), 8.82 (d, *J* = 8.2 Hz, H^3′^ bipy^2^, 1 H), 8.75 (d, *J* = 8.1 Hz, H^3^ bipy^2^, 1 H), 8.57 (d, *J* = 5.6 Hz, H^6′^ bipy^2^, 1 H), 8.49 (s, H^3^ Indz, 1 H), 8.29 (td, *J* = 8.0, 1.5 Hz, H^4′^ bipy^2^, 1 H), 8.26–8.19 (m, H^4^ bipy^1^ and H^4′^ bipy^1^, 2 H), 8.17–8.09 (m, H^6^ bipy^1^, H^4^ bipy^2^ and *o*-C_6_H_5_, 3 H), 8.02 (d, *J* = 6.4 Hz, H^6^ bipy^2^, 1 H), 7.85–7.76 (m, H^4^ indz, *o*-C_6_H_5_, *m*-C_6_H_5_ and *p*-C_6_H_5_, 4 H), 7.72 (dddd, *J* = 9.1, 7.3, 5.7, 1.3 Hz, H^5′^ bipy^1^ and H^5′^ bipy^2^, 2 H), 7.66 (td, *J* = 7.5, 1.5 Hz, *m*-C_6_H_5_, 1 H), 7.60 (ddd, *J* = 7.5, 5.6, 1.3 Hz, H^5^ bipy^1^, 1 H), 7.50 (ddd, *J* = 7.3, 5.7, 1.3 Hz, H^5^ bipy^2^, 1 H), 7.45–7.39 (m, H^5^ indz and H^6^ indz, 2 H), 6.66 (s, H^7^ indz, 1 H). ^13^C NMR (126 MHz, acetone, 298 K): δ 164.47 (1C, NH=*C*Ph), 157.75 (1C, C^2^ bipy^2^), 157.50 (1C, C^2^ bipy^1^), 157.44 (1C, C^2′^ bipy^2^), 157.43 (1C, C^2′′^ bipy^1^), 153.55 (1C, C^6′^ bipy^1^), 152.12 (1C, C^6^ bipy^2^), 152.05 (1C, C^6^ bipy^1^), 152.02 (1C, C^6′^ bipy^2^), 143.76 (1C, C^3^ indz), 140.39 (1C, C^7a^ indz), 138.05 (1C, C^4′^ bipy^2^), 137.97 (1C, C^4′^ bipy^1^), 137.84 (1C, C^4^ bipy^1^), 137.65 (1C, C^4^ bipy^2^), 132.73 (1C, *p*-C_6_H_5_), 129.69 (1C, C^6^ indz), 129.21 (1C, *ipso*-C_6_H_5_), 127.92 (1C, C^5′^ bipy^2^), 127.77 (1C, C^5′^ bipy^1^), 127.30 (1C, C^5^ bipy^1^), 126.91 (2C, C^5^ bipy^2^ and C^3a^ indz), 124.95 (1C, C^5^ indz), 124.20 (1C, C^3^ bipy^1^), 124.11 (1C, C^3′^ bipy^1^), 123.84 (1C, C^3′^ bipy^2^), 123.73 (1C, C^3′^ bipy^1^), 121.70 (1C, C^4^ indz), 111.97 (1C, C^7^ indz). ^13^C NMR (126 MHz, acetone, 243 K): δ 164.47 (1C, NH=*C*Ph), 157.75 (1C, C^2^ bipy^2^), 157.50 (1C, C^2^ bipy^1^), 157.44 (1C, C^2′^ bipy^2^), 157.43 (1C, C^2′^ bipy^1^), 153.55 (1C, C^6′^ bipy^1^), 152.12 (1C, C^6^ bipy^2^), 152.05 (1C, C^6^ bipy^1^), 152.02 (1C, C^6′^ bipy^2^), 143.76 (1C, C^3^ indz), 140.39 (1C, C^7a^ indz), 138.05 (1C, C^4′^ bipy^2^), 137.97 (1C, C^4′^ bipy^1^), 137.84 (1C, C^4^ bipy^1^), 137.65 (1C, C^4^ bipy^2^), 132.73 (1C, *p*-C_6_H_5_), 129.86 (1C, *m*-C_6_H_5_), 129.69 (1C, C^6^ indz), 129.45 (1C, *o*-C_6_H_5_), 129.21 (1C, *ipso*-C_6_H_5_), 129.08 (1C, *m*-C_6_H_5_), 128.53 (1C, *o*-C_6_H_5_), 127.92 (1C, C^5′^ bipy^2^), 127.77 (1C, C^5′^ bipy^1^), 127.30 (1C, C^5^ bipy^1^), 126.91 (2C, C^5^ bipy^2^ and C^3a^ indz), 124.95 (1C, C^5^ indz), 124.20 (1C, C^3^ bipy^1^), 124.11 (1C, C^3′^ bipy^1^), 123.84 (1C, C^3′^ bipy^2^), 123.73 (1C, C^3′^ bipy^1^), 121.70 (1C, C^4^ indz), 111.97 (1C, C^7^ indz). ^15^N NMR (51 MHz, acetone-*d*_6_): δ −119.99 (1N, N^2^ indz), −129.42 (1N, N^1′^ bipy^1^), −130.56 (1N, N^1′^ bipy^2^), −131.53 (1N, N^1^ bipy^1^), −134.04 (1N, N^1^ bipy^2^), −167.93 (1N, N^1^ indz), −176.81 (1N, NH). IR (solid, cm^–1^): 3508 w, 3169 w, 3156 w, 3085 w, 1710 w, 1606 w, 1571 w, 1493 m, 1467 m, 1442 m, 1352 w, 1333 w, 1313 w, 1253 vs, 1225 vs, 1155 s, 1125 s, 1100 s, 1071 m, 1028 m, 1028 vs, 919 w, 892 w, 796 w, 765 s, 732 m, 716 m, 701 m, 664 w, 634 vs. Anal. Calcd for C_36_H_27_F_6_N_7_O_6_RuS_2_: C, 46.35; H, 2.92; N, 10.51; S, 6.88. Found: C, 46.53; H, 3.13; N, 10.85; S, 7.10.

### *cis*-[Ru(bipy)_2_(NH=C(Ph)dmpz-κ^2^*N*,*N*)](OTf)_2_ (**4c**)

The same procedure as for **4a**, using **2c** (0.083 g, 0.1 mmol) as the starting material, gave 0.073 g (80%) of **4c** as a red microcrystalline solid. ^1^H NMR (500 MHz, acetone-*d*_6_, 298 K): δ 11.45 (s, NH, 1 H), 8.98 (ddd, *J* = 5.6, 1.5, 0.7 Hz, H^6′^ bipy^1^, 1 H), 8.86 (dt, *J* = 8.3, 1.2 Hz, H^3′^ bipy^1^, 1 H), 8.84 (dt, *J* = 8.2, 1.2 Hz, H^3^ bipy^1^, 1 H), 8.79 (dt, *J* = 8.2, 1.2 Hz, H^3′^ bipy^2^, 1 H), 8.68 (dt, *J* = 8.2, 1.2 Hz, H^3^ bipy^2^, 1 H), 8.47 (ddd, *J* = 5.6, 1.5, 0.8 Hz, H^6′^ bipy^2^, 1 H), 8.29 (tdd, *J* = 8.2, 1.5, 0.6 Hz, H^4′^ bipy^1^ and H^4′^ bipy^2^, 2 H), 8.17 (ddd, *J* = 8.1, 7.6, 1.5 Hz, H^4^ bipy^1^, 1 H), 8.12 (ddd, *J* = 5.6, 1.5, 0.8 Hz, H^6^ bipy^1^, 1 H), 8.05 (m, H^4^ bipy^2^ and *o*-C_6_H_5_, 2 H), 7.86–7.80 (m, H^6^ bipy^2^ and H^5′^ bipy^1^, 2 H), 7.79 (ddd, *J* = 7.7, 5.6, 1.3 Hz, H^5′^ bipy^2^, 1 H), 7.68 (m, *m*-C_6_H_5_ and *p*-C_6_H_5_, 2 H), 7.56 (m, H^5^ bipy^1^, *o*-C_6_H_5_ and *m*-C_6_H_5_, 3 H), 7.42 (ddd, *J* = 7.6, 5.7, 1.3 Hz, H^5^ bipy^2^, 1 H), 6.46 (s, H^4^ dmpz, 1 H), 1.95 (s, CH_3_^5^ dmpz, 3 H), 1.70 (s, CH_3_^3^ dmpz, 3 H). dmpz, 1 H), 1.95 (s, C^5^H_3_ dmpz, 3 H), 1.70 (s, C^3^H_3_ dmpz, 3 H). ^1^H NMR (500 MHz, acetone-*d*_6_, 243 K): δ 11.55 (s, NH, 1 H), 8.98 (dt, *J* = 5.4, 1.1 Hz, H^6′^ bipy^1^, 1 H), 8.89 (ddt, *J* = 10.2, 8.2, 1.0 Hz, H^3′^ bipy^1^, 1 H), 8.83 (dt, *J* = 8.2, 1.1 Hz, H^3^ bipy^1^, 1 H), 8.73 (dt, *J* = 8.2, 1.0 Hz, H^3′^ bipy^2^, 1 H), 8.69 (dt, *J* = 8.2, 1.2 Hz, H^3^ bipy^2^, 1 H), 8.49 (ddd, *J* = 5.6, 1.5, 0.8 Hz, H^6′^ bipy^2^, 1 H), 8.30 (tt, *J* = 7.9, 1.4 Hz, H^4′^ bipy^1^ and H^4′^ bipy^2^, 2 H), 8.19 (td, *J* = 7.8, 1.5 Hz, H^4^ bipy^1^, 1 H), 8.14 (ddd, *J* = 5.6, 1.5, 0.7 Hz, H^6^ bipy^1^, 1 H), 8.11 (dt, *J* = 7.7, 1.9 Hz, *o*-C_6_H_5_, 1 H), 8.07 (td, *J* = 7.9, 1.5 Hz, H^4^ bipy^2^, 1 H), 7.87–7.66 (m, H^6^ bipy^2^ and H^5′^ bipy^1^, 2 H), 7.79 (ddd, *J* = 7.7, 5.6, 1.3 Hz, H^5′^ bipy^2^, 1 H), 7.71–7.66 (m, *m*-C_6_H_5_ and *p*-C_6_H_5_, 2 H), 7.57 (ddd, *J* = 10.8, 9.5, 7.6, 6.3 Hz, H^5^ bipy^1^ and *o*-C_6_H_5_ and *m*-C_6_H_5_, 3 H), 7.44 (ddd, *J* = 7.6, 5.7, 1.3 Hz, H^5^ bipy^2^, 1 H), 6.49 (s, H^4^ dmpz, 1 H), 1.95 (s, C^5^H_3_ dmpz, 3 H), 1.67 (s, C^3^H_3_ dmpz, 3 H). ^13^C NMR (126 MHz, acetone, 298 K): δ 165.46 (1C, NH=*C*Ph), 158.13 (1C, C^2^ bipy^2^), 157.75 (1C, C^3^ dmpz), 157.53 (1C, C^2′^ bipy^2^), 157.48 (1C, C^2^ bipy^1^), 157.37 (1C, C^2′^ bipy^1^), 153.06 (1C, C^6′^ bipy^1^), 152.37 (1C, C^6′^ bipy^2^), 152.11 (1C, C^6^ bipy^2^), 151.83 (1C, C^6^ bipy^1^), 147.19 (1C, C^5^ dmpz), 137.79 (1C, C^4′^ bipy^2^), 137.65 (1C, C^4′^ bipy^1^), 137.60 (1C, C^4^ bipy^1^), 137.47 (1C, C^4^ bipy^2^), 132.12 (1C, *p*-C_6_H_5_), 129.97 (1C, *ipso*-C_6_H_5_), 128.04 (1C, C^5′^ bipy^2^), 127.85 (1C, C^5′^ bipy^1^), 127.54 (1C, C^5^ bipy^1^), 126.91 (1C, C^5^ bipy^2^), 124.36 (1C, C^3^ bipy^1^), 124.20 (1C, C^3′^ bipy^1^), 123.87 (1C, C^3′^ bipy^2^), 123.71 (1C, C^3^ bipy^2^), 114.03 (1C, C^4^ dmpz), 13.72 (1C, C^5^H_3_ dmpz), 11.50 (1C, C^3^H_3_ dmpz). ^13^C NMR (126 MHz, acetone, 243 K): δ 165.26 (1C, NH=*C*Ph), 158.07 (1C, C^2^ bipy^2^), 157.55 (1C, C^3^ dmpz), 157.45 (1C, C^2′^ bipy^2^), 157.30 (1C, C^2^ bipy^1^), 157.21 (1C, C^2′^ bipy^1^), 153.18 (1C, C^6′^ bipy^1^), 152.57 (1C, C^6′^ bipy^2^), 152.23 (1C, C^6^ bipy^2^), 151.99 (1C, C^6^ bipy^1^), 147.02 (1C, C^5^ dmpz), 137.80 (1C, C^4′^ bipy^2^), 137.65 (1C, C^4′^ bipy^1^), 137.52 (1C, C^4^ bipy^1^), 137.47 (1C, C^4^ bipy^2^), 132.17 (1C, *p*-C_6_H_5_), 130.02 (1C, *ipso*-C_6_H_5_), 129.35 (1C, *m*-C_6_H_5_), 129,12 (1C, *o*-C_6_H_5_), 128.83 (1C, *m*-C_6_H_5_), 128.66 (1C, *o*-C_6_H_5_), 128.11 (1C, C^5′^ bipy^2^), 127.94 (1C, C^5′^ bipy^1^), 127.64 (1C, C^5^ bipy^1^), 127.02 (1C, C^5^ bipy^2^), 124.42 (1C, C^3^ bipy^1^), 124.26 (1C, C^3′^ bipy^1^), 123.84 (1C, C^3′^ bipy^2^), 123.69 (1C, C^3^ bipy^2^), 113.86 (1C, C^4^ dmpz), 13.98 (1C, C^5^H_3_ dmpz), 11.54 (1C, C^3^H_3_ dmpz). ^15^N NMR (51 MHz, acetone-*d*_6_): δ −127.69 (1N, N^1^ bipy^1^), −128.28 (1N, N^1′^ bipy^1^), −130.15 (1N, N^1′^ bipy^2^), −134.67 (1N, N^1^ bipy^2^), −143.09 (1N, N^2^ dmpz), −152.63 (1N, N^1^ dmpz), −167.52 (1N, NH). IR (solid, cm^–1^): 3576 w, 3513 m, 3189 m, 2114 m, 2083 m, 2937 w, 2324 w, 2164 w, 2051 w, 1981 w, 1903 w, 1635 w, 1601 m, 1581 w, 1565 m, 1496 w, 1466 m, 1445 s, 1420 s, 1347 w, 1252 vs, 1225 vs, 1145 vs, 1110 s, 1057 m, 1028 vs, 999 m, 944 m, 932 m, 899 w, 841 w, 803 w, 767 vs, 732 s, 705 m, 677 w, 661 w, 631 vs. Anal. Calcd for C_29_H_27_F_6_N_7_O_6_RuS_2_: C, 44.84; H, 3.21; N, 10.76; S, 7.04. Found: C, 44.53; H, 3.23; N, 10.92; S, 7.20.

### *cis*-[Ru(bipy)_2_(NH=C(p-Tol)pz-κ^2^*N*,*N*)](OTf)_2_ (**5**)

The same procedure as for **4a**, using *p*-tolunitrile (0.023 g, 0.2 mmol) as the nitrile, gave 0.064 g (72%) of **5** as a red microcrystalline solid. ^1^H NMR (500 MHz, acetone-*d*_6_): δ 11.63 (s, NH, 1 H), 8.92 (d, *J* = 5.0 Hz, H^6′^ bipy^1^, 1 H), 8.80–8.68 (m, H^3^ bipy^1^, H^3^ bipy^2^, H^3′^ bipy^1^ and H^3′^ bipy^2^, 4 H), 8.65 (dd, *J* = 3.3, 0.6 Hz, H^5^ pz, 1 H), 8.27–8.23 (m, H^6′^ bipy^2^, H^4′^ bipy^1^ and H^4′^ bipy^2^, 3 H), 8.20–8.15 (m, H^4^ bipy^1^, 1 H), 8.14–8.09 (m, H^4^ bipy^2^, 1 H), 8.07 (ddd, *J* = 5.6, 1.5, 0.8 Hz, H^6^ bipy^1^, 1 H), 8.02–7.99 (m, H^6^ bipy^2^, 1 H), 7.86 (d, *J* = 8.3 Hz, *o*-C_6_H_5_, 2 H), 7.82 (d, *J* = 1.9 Hz, H^3^ pz, 1 H), 7.78–7.68 (m, H^5′^ bipy^1^, H^5′^ bipy^2^ 2 H), 7.64 (d, *J* = 8.1 Hz, H^5^ bipy^1^, 1 H), 7.54–7.43 (m, *meta*-C_6_H_5,_2 H), 7.38 (d, *J* = 8.6 Hz, H^5^ bipy^2^, 1 H), 6.92 (dd, *J* = 3.2, 2.1 Hz, H^4^ pz, 1 H), 2.45 (s, CH_3_, 1H). ^13^C NMR (126 MHz, acetone-*d*_6_): δ 164.33 (1C, NH=*C*PhCH_3_), 158.03 (1C, C^2^ bipy^1^), 157.49 (1C, C^2^ bipy^2^), 157.49 (1C, C^2′^ bipy^1^), 157.28 (1C, C^2′^ bipy^2^), 153.06 (1C, C^6′^ bipy^1^), 152.39 (1C, C^6^ bipy^1^), 151.97 (1C, C^6^ bipy^2^), 151.67 (1C, C^6′^ bipy^2^), 146.09 (1C, C^3^ pz), 138.31 (1C, C^4′^ bipy^2^), 138.02 (1C, C^4′^ bipy^1^), 137.72 (1C, C^4^ bipy^1^), 137.61 (1C, C^4^ bipy^2^), 135.49 (1C, C^5^ pz), 130.02 (2C, *m*-C_6_H_5_), 128.80 (1C, *ipso*-C_6_H_5_), 128.92 (2C, *o*-C_6_H_5_), 128.12 (1C, C^5′^ bipy^2^), 127.81 (1C, C^5′^ bipy^1^), 127.30 (1C, C^5^ bipy^1^), 127.00 (1C, C^5^ bipy^2^), 124.47 (1C, C^3′^ bipy^1^), 124.00 (1C, C^3′^ bipy^2^), 123.95 (1C, C^3^ bipy^2^), 123.84 (1C, C^3^ bipy^1^), 112.03 (1C, C^4^ pz), 21.02 (1C, CH_3_). IR (solid, cm^–1^): 3326 m, 3083 w, 2860 w, 2324 w, 2287 w, 2164 w, 2140 w, 2113 w, 2080 w, 2051 w, 1981 w, 1660 w, 1633 w, 1606 w, 1465 m, 1422 s, 1244 m, 1225 m, 1161 m, 1124 m, 1085 m, 1050 m, 1030 m, 957 m, 888 m, 831 vs, 759 vs, 728 vs, 636 m. Anal. Calcd for C_33_H_27_F_6_N_7_O_6_RuS_2_: C, 44.19; H, 3.04; N, 10.94; S, 7.15. Found: C, 44.22; H, 3.24; N, 12.87; S, 7.33.

### *cis*-[Ru(bipy)_2_(NH=C(p-FC_6_H_4_)pz-κ^2^*N*,*N*)](OTf)_2_ (**6**)

The same procedure as for **4a**, using 4-fluorobenzonitrile (0.024 g, 0.2 mmol) as the nitrile, gave 0.060 g (66%) of **6** as a red microcrystalline solid. ^1^H NMR (500 MHz, acetone-*d*_6_): δ 11.75 (s, NH, 1 H), 8.94 (d, *J* = 5.6 Hz, H^6′^ bipy^1^, 1 H), 8.80 (dd, *J* = 8.1, 1.1 Hz, H^3′^ bipy^1^, 1 H), 8.78–8.74 (m, H^3^ bipy^1^ and H^3′^ bipy^2^, 2 H), 8.69 (d, *J* = 7.5 Hz, H^3^ bipy^2^, 1 H), 8.66 (d, *J* = 3.2 Hz, H^5^ pz, 1 H), 8.30–8.22 (m, H^6′^ bipy^2^, H^4′^ bipy^1^ and H^4′^ bipy^2^, 3 H), 8.17 (td, *J* = 7.8, 1.2 Hz, H^4^ bipy^1^, 1 H), 8.13–8.01 (m, H^6^ bipy^1^, H^4^ bipy^2^ and *o*-C_6_H_5_, 3 H), 7.99 (d, *J* = 4.4 Hz, H^6^ bipy^2^, 1 H), 7.81 (d, *J* = 2.1 Hz, H^3^ pz, 1 H), 7.76–7.68 (m, H^5′^ bipy^1^, H^5^ bipy^2^, H^5′^ bipy^2^, 3H), 7.54 (ddd, *J* = 7.4, 5.6, 1.3 Hz, H^5^ bipy^1^, 1 H), 7.51–7.39 (m, *m*-C_6_H_5_, 2 H), 6.90 (dd, *J* = 3.2, 2.2 Hz, H^4^ pz, 1 H). ^13^C NMR (126 MHz, acetone-*d*_6_): δ 163.10 (1C, NH=*C*PhF), 158.27 (1C, C^2^ bipy^2^), 157.54 (1C, C^2^ bipy^1^), 157.54 (1C, C^2′^ bipy^1^), 157.28 (1C, C^2′^ bipy^2^), 153.58 (1C, C^6′^ bipy^1^), 152.34 (1C, C^6^ bipy^2^), 152.11 (1C, C^6^ bipy^1^), 151.74 (1C, C^6′^ bipy^2^), 146.32 (1C, C^3^ pz), 138.15 (1C, C^4′^ bipy^2^), 137.92 (1C, C^4′^ bipy^1^), 137.62 (1C, C^4^ bipy^1^), 137.69 (1C, C^4^ bipy^2^), 135.52 (1C, C^5^ pz), 131.91 (2C, *o*-C_6_H_5_), 131.97 (1C, *ipso*-C_6_H_5_), 127.86 (1C, C^5^ bipy^2^), 127.56 (1C, C^5′^ bipy^1^), 127.33 (1C, C^5^ bipy^1^), 127.10 (1C, C^5′^ bipy^2^), 124.35 (1C, C^3′^ bipy^1^), 124.10 (1C, C^3′^ bipy^2^), 123.82 (1C, C^3^ bipy^1^), 123.78 (1C, C^3^ bipy^2^), 116.66 (2C, *m*-C_6_H_5_), 112.24 (1C, C^4^ pz). IR (solid, cm^–1^): 3322 m, 3085 w, 2862 w, 2324 w, 2163 w, 2141 w, 2113 w, 2050 w, 1981 w, 1658 w, 1606 w, 1530 w, 1465 m, 1423 s, 1243 m, 1161 m, 1126 m, 1048 m, 1028 m, 993 m, 829 vs, 758 s, 740 s, 716 s, 637 m. Anal. Calcd for C_32_H_24_F_8_N_7_O_6_RuS_2_: C, 42.67 H, 2.69; N, 10.89; S, 7.12. Found: C, 42.88; H, 2.98; N, 10.52; S, 7.35.

### General Procedure for the Oxidation of Sulfides

A glass vial was loaded with the corresponding sulfide **7a**–**g** (0.2 mmol), and complex **3c** (0.002 mmol, 1 mol %). Then, 2.0 mL of absolute ethanol was added, and the reaction mixture was stirred without exclusion of air under irradiation of a white LED system (see the Supporting Information). After 1 h of stirring at rt, the crude reaction mixture was filtered over a plug of Celite/silica gel to afford the corresponding pure sulfoxide **8a**–**g**.

#### Methyl 4-Methylphenyl Sulfoxide (**8a**).^26^

Colorless oil (29.5 mg, 98% yield). ^1^H NMR (300 MHz, CDCl_3_): δ 7.51 (d, *J* = 8.3 Hz, H^2^ and H^6^ Ar, 2H), 7.30 (d, *J* = 8.3 Hz, H^3^ and H^5^ Ar, 2H), 2.67 (s, CH_3_-SO, 3H), 2.38 (s, CH_3_-Ar, 3H).

#### Benzyl Phenyl Sulfoxide (**8b**).^26^

Beige solid (39.9 mg, 92% yield). ^1^H NMR (300 MHz, CDCl_3_): δ 7.39–7.29 (m, Ph-SO, 5H), 7.22–7.15 (m, Ph of Bn, 3H), 6.92–6.89 (m, Ph of Bn, 2H), 4.02 (d, *J* = 12.6 Hz, CH_2_, 1H), 3.92 (d, *J* = 12.6 Hz, CH_2_, 1H).

#### Allyl Phenyl Sulfoxide (**8c**).^26^

Colorless oil (29.7 mg, 90% yield). ^1^H NMR (300 MHz, CDCl_3_): δ 7.61–7.58 (m, H^2^ and H^6^ Ar, 2H), 7.51–7.49 (m, H^3^, H^5^ and H^4^ Ar, 3H), 5.71–5.57 (m, CH=, 1H), 5.33 (d, *J* = 9.8 Hz, CH_2_=, 1H), 5.19 (d, *J* = 16.0 Hz, CH_2_=, 1H), 3.46–3.60 (m, CH_2_, 2H).

#### Dibutyl Sulfoxide (**8d**).^26^

Colorless oil (24.4 mg, 75% yield). ^1^H NMR (300 MHz, CDCl_3_): δ 2.75–2.55 (m, CH_2_-SO, 4H), 1.85–1.65 (m, C*H*_2_-CH_2_-SO, 4H), 1.63–1.32 (m, C*H*_2_-CH_2_-CH_2_-SO, 4H), 0.97 (t, *J* = 7.3 Hz, CH_3_, 6H).

#### (*tert*-Butyl)methylsulfoxide (**8e**).^26^

Colorless oil (18.6 mg, 78% yield). ^1^H NMR (300 MHz, CDCl_3_): δ 2.36 (s, CH_3_-SO, 3H), 1.24 (s, *t*Bu, 9H).

### 2-[(Diphenylmethyl)sulfinyl]acetamide, Modafinil (**8f**).^40^

The crude mixture was purified by flash chromatography (dichloromethane/methanol 10/1 as eluent) to afford **8f** as a white solid (45.6 mg, 79% yield). ^1^H NMR (300 MHz, CDCl_3_): δ 7.50 (dd, *J* = 9.1, 3.0 Hz, H^4^ Ph, 2H), 7.43–7.36 (m, H^2^, H^6^, H^3^, and H^5^ Ph, 8H), 7.03 (br s, NH_2_, 1H), 5.55 (br s, NH_2_, 1H), 5.10 (s, Ph_2_CH, 1H), 3.49 (d, CH_2_, *J* = 14.9 Hz, 1H), 3.09 (d, CH_2_, *J* = 14.9 Hz, 1H).

### Photophysical Experiments

The solvents used for the spectroscopic studies were of spectroscopic grade and were used as received. Fluorescence and ultraviolet–visible (UV–vis) spectra were recorded in optically dilute solutions (from 10^–5^ to 5 × 10^–5^ M), at rt with a quartz cuvette (1 cm × 1 cm), using Hitachi U-3900 and F-7000 fluorescence spectrophotometers, respectively. Fluorescence decay lifetimes were determined in deaerated solvents, using a time-correlated single photon counting instrument (FLS980 Series, Edinburgh instruments) with a 405 nm pulsed LED (Edinburgh instruments, EPL-510) light source having a 50–500 ns pulse. In each solvent the absolute fluorescence quantum yields were obtained using an Edinburgh FLS980 Series instrument with a integrating sphere accessory, using the solvent as a reference. χ^2^ is a statistical parameter that accounts for the quality of fit between the model exponential decays and the observed (ideal value = 1). The FAST software package (Edinburgh Instruments) was used to obtain tail fits and numerical reconvolution.

### Electrochemical Experiments

Electrochemical experiments were performed in a three-electrode cell with one cavity, using a platinum=disk working electrode (ø = 3 mm), a platinum-wire counter electrode (ø = 0.5 mm), and a saturated calomel electrode (SCE) reference electrode in acetonitrile. All of the potentials given are referenced to the SCE electrode. The redox properties of all the complexes were studied with cyclic voltammetry (CV) at different scan rates in a 0.1 M solution (nBu_4_N)(PF_6_) (TBAH) in MeCN, and their redox potentials are also referenced to the SCE electrode.

### Crystal Structure Determination for Compounds **3a**–**c** and **4a**–**c**

Crystals were grown by prolonged diffusion of Et_2_O into concentrated solutions of the compounds in acetonitrile (for **3a**–**c**) or acetone (for **4a**–**c**) at −20 °C. All crystallographic details can be found in the CIF files. A crystal was adhered to a glass fiber and fixed to an Agilent SuperNova diffractometer fitted with an Atlas CCD detector. The crystals were maintained at 293(2) K during data collection. The structures were solved using Olex2,^41^ with the ShelXT^42^ structure solution program. The structures were then refined with the ShelXL^43^ refinement package using least-squares minimization. All non-hydrogen atoms were refined anisotropically, whereas hydrogen atoms were fixed in calculated positions and refined with a common thermal parameter as riding atoms. All graphics were made with Olex2, and distances and angles of hydrogen bonds were calculated with PARST^44,45^ (normalized values).^46,47^

## Results and Discussion

### Syntheses and Characterization of the Complexes

**Scheme 2:**
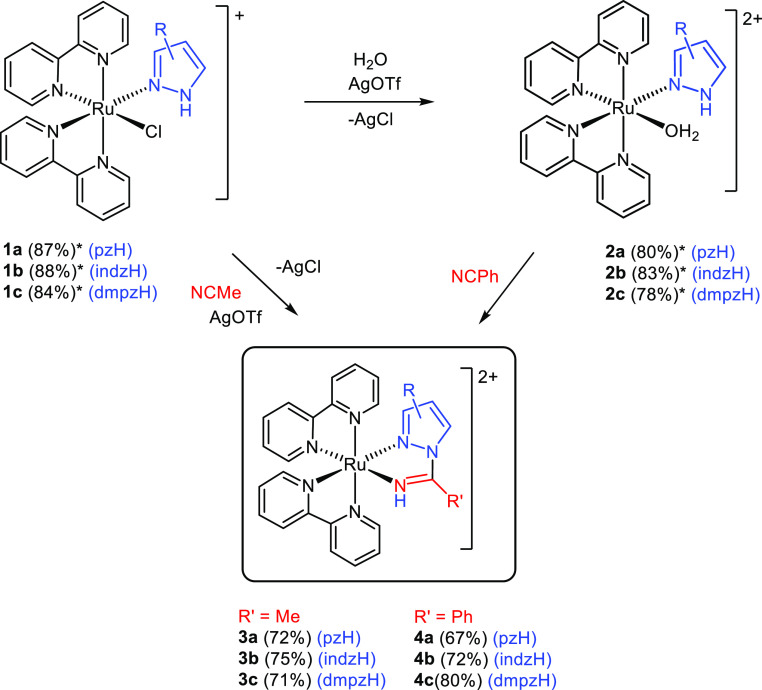
Synthesis of the New 1,2-Azolylamidino Complexes Yields are given in parentheses; those with an asterisk are reported in ref (39).

Complexes with varied substituents were obtained in order to confirm the synthetic method, as well as to determine the effect of the substituents on the properties of the 1,2-azolylamidino complexes obtained. The 1,2-azolylamidino ligands are the result of the coupling of pyrazole (pzH), indazole (indzH), or 3,5-dimethylpyrazole (dmpzH) with either acetonitrile (MeCN) or benzonitrile (PhCN). All of the complexes described in this work are collected in Table 1 and Scheme 2. In addition to the 1,2-azolylamidino complexes, Table 1 and Scheme 2 include the mixed 1,2-azole–chlorido complexes *cis*-[Ru(bipy)_2_Cl(az*H)]OTf (**1**) and the 1,2-azole–aquo complexes *cis*-[Ru(bipy)_2_(H_2_O)(az*H)](OTf)_2_ (**2**) previously reported by us^39^ and used in this work as starting materials.

**Table 1:** Complexes Used in This Study

	pzH	indzH	dmpzH	ref
*cis*-[Ru(bipy)_2_Cl(az*H)]OTf	**1a**	**1b**	**1c**	(39)
*cis*-[Ru(bipy)_2_(H_2_O)(az*H)](OTf)_2_	**2a**	**2b**	**2c**	(39)
*cis*-[Ru(bipy)_2_(NH=C(Me)az*-κ^2^*N*,*N*)](OTf)_2_	**3a**	**3b**	**3c**	this work
*cis*-[Ru(bipy)_2_(NH=C(Ph)az*-κ^2^*N*,*N*)](OTf)_2_	**4a**	**4b**	**4c**	this work

Chlorido–1,2-azole complexes **1** lead to 1,2-azolylamidino complexes **3**, after the chlorido ligand is removed with silver triflate in the presence of MeCN. The 1,2-azolylamidino complexes derived from PhCN are better obtained after removal of the chlorido ligand from **1** in the presence of H_2_O to generate the aquo complexes **2**,^39^ which lead to the 1,2-azolylamidino complexes **4** after addition of PhCN. A catalytic amount of NaOH(aq) was added in order to obtain the 1,2-azolylamidino complexes with pz (**4a**) and dmpz (**4c**), whereas no base was needed in order to obtain the indazolylamidino complex (**4b**). This fact is in agreement with the higher acidity of indzH in comparison to pzH or dmpzH.^38^ The 1,2-azolylamidino complexes with PhCN (**4**) can also be obtained from the chlorido–1,2-azole precursors **1**, by adding PhCN instead of MeCN. However, this route gives lower yields.

**Figure 2:**
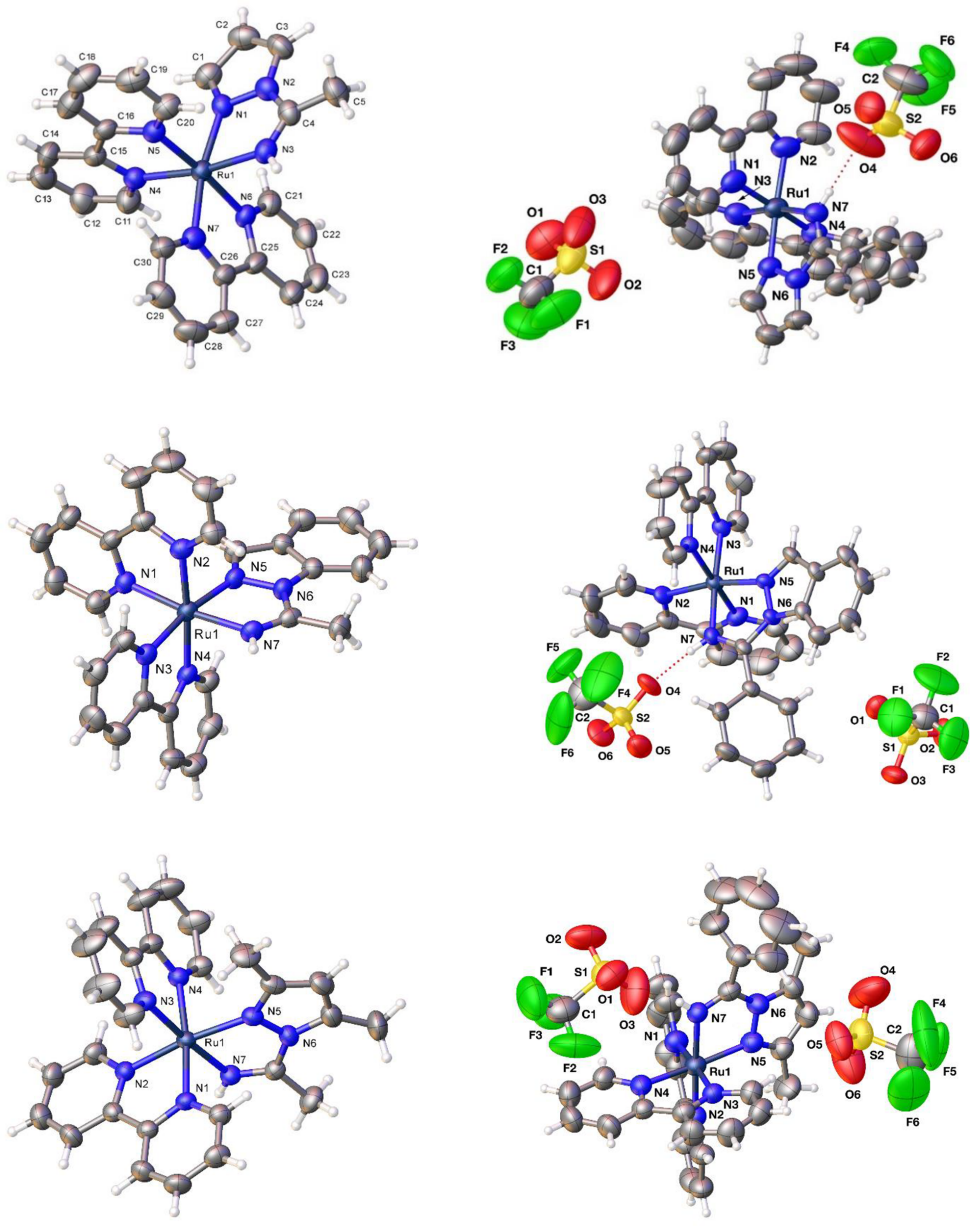
Perspective views of **3a**–**3c** (left from top to bottom, anions not shown) and **4a**–**c** (right from top to bottom, showing triflate anions) showing the atom numbering. Thermal ellipsoids are drawn at 50% probability.

Complexes **3** and **4** were all characterized by single-crystal X-ray diffractometry (Figure 2). The distances and angles (CCDC 2044577–2044582) are analogous to those found in other 1,2-azolylamidino ruthenium(II) complexes.^48−52^ In complexes **3b**,**c** and **4a**–**c**, the *N*-bound hydrogens of the 1,2-azolylamidino ligands are involved in hydrogen bonding with an oxygen atom of a OTf^–^ anion. The distances and angles detected for **3b** (H(7)···O(2) 1.99(6) Å, N(7)···O(2) 2.973(1) Å, N(7)–H(7)···O(2) 159.6(3)°), **3c** (H(7)···O(3) 1.92(2) Å, N(7)···O(3) 2.842(17) Å, N(7)–H(7)···O(3) 149.7(5)°), **4a** (H(7)···O(4) 1.89(2) Å, N(7)···O(4) 2.831(19) Å, N(7)–H(7)···O(4) 150.6(6)°), **4b** (H(7)···O(4) 2.032(4) Å, N(7)···O(4) 2.821(6) Å, N(7)–H(7)···O(4) 149.8(3)°), and **4c** (H(7)···O(3) 2.00(9) Å, N(7)···O(3) 2.941(9) Å, N(7)–H(7)···O(3) 151(8)°) may be considered as “moderate” hydrogen bonds.^53,54^

The spectroscopic and analytical data of 1,2-azolylamidino complexes **3** and **4** are collected in the Experimental Section and sustain the proposed geometries. All the ^1^H NMR and ^13^C NMR spectra are displayed in the Supporting Information (Figures S1–S16). Their ^1^H, ^13^C, and ^15^N NMR spectra show the expected signals. The hydrogen atoms of the phenyl groups in benzonitrile-derived complexes **4b**,**c** show broad signals in the ^1^H NMR and ^13^C NMR spectra at room temperature, which may be explained by considering the slow rotation of the phenyl group. *ortho* and *meta* protons and carbons both become respectively inequivalent due to this slow motion. Spectra recorded at low temperatures gave the expected pattern with sharp signals (Figures S17 and S18). On the other hand, the NMR spectra of **4a** (where the substituent is pz, smaller than indz and dmpz) display well-defined signals where each *ortho* and each *meta* proton and carbon are equivalents at rt, which indicates that the phenyl group in the pyrazolylamidino ligand freely rotates in this complex.

### Photophysical Studies

**Figure 3:**
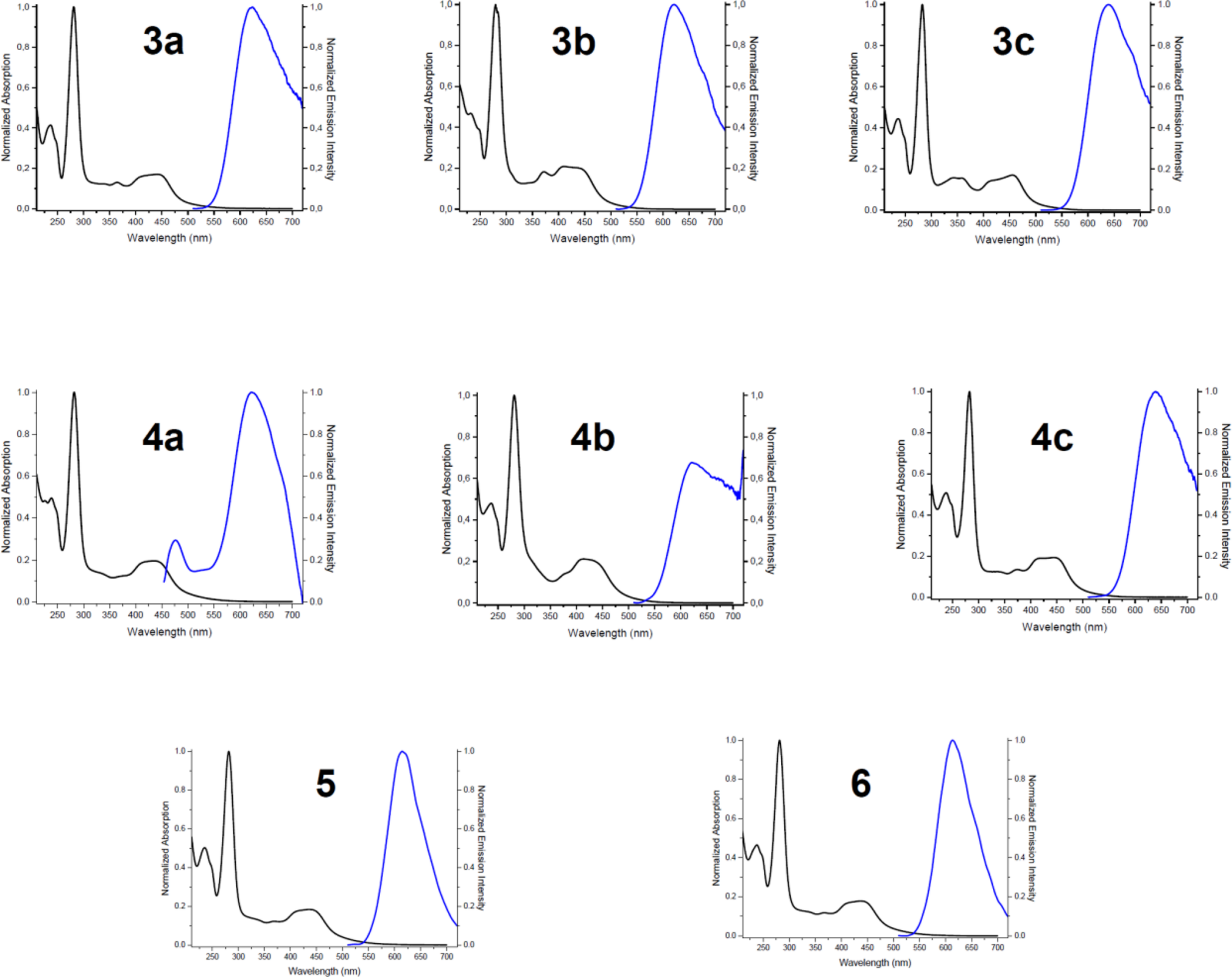
Normalized UV/vis absorption (black) and emission (blue, λ_ex_ = 420 nm) spectra, in deaerated solvents in optically dilute MeCN solutions at 298 K.

The absorption and emission spectral data for all of the complexes herein reported are collected in Tables 2 and 3. The data for the previously described complexes **1**(39) are also included in both tables for comparison purposes. The absorption and emission spectra (Figure 3) and the wavelength maxima observed at 298 K in different deaerated solvents are summarized in Figure S19 in the Supporting Information. The spectra of complexes **3** and **4** show absorption patterns analogous to those previously described for similar complexes.^55−64^ In the 250–300 nm region all of the 1,2-azolylamidino complexes exhibit intense absorption bands that may be ascribed to π(L) → π*(L) intraligand transitions (IL), whereas the broad lower energy bands above 300 nm correspond to dπ(Ru) → π*(L) metal to ligand charge transfer bands (MLCT). The low-energy bands of all the complexes are blue-shifted when the chlorido–1,2-azole ligands in **1** are substituted by a 1,2-azolylamidino ligand, and these blue shifts are due to electronic effects caused by the substitution of the electron-donating, anionic chlorido ligand by the σ-donating, neutral amidino ligand. A similar behavior was observed for complexes **1** when the chlorido ligand was replaced by an aquo ligand to generate complexes **2**.^39^ The MLCT band can be easily shifted or tuned depending on the 1,2-azolylamidino substituents, as can be easily concluded from the values collected in Table 2. For example, a comparison of the lower energy bands between complexes derived from the same nitrile leads to the same secuence: indz (**c**) > pz (**a**) > dmpz (**b**) for both complexes **3** (obtained from MeCN) and **4** (obtained from PhCN).

**Table 2:** Absorption and Emission Data of Complexes **1** and **3**–**6** in MeCN

compd	absorption λ (nm) (ε (10^–3^ M^–1^cm^–1^))	emission λ_em_ (nm) (λ_excit_ = 420 nm)	ref
**1a**	237 (19.9), 287 (49.5), 341 (7.30), 477 (7.30)	625	(39)
**1b**	236 (24.1), 287 (54.9), 338 (8.10), 476 (8.30)	646	(39)
**1c**	236 (23.5), 287 (55.8), 341 (8.60), 473 (8.50)	640	(39)
**3a**	237 (23.3), 281 (56.4), 365 (7.27), 442 (9.51).	622	this work
**3b**	232 (40.1), 280 (85.8), 373 (15.6), 410 (17.8)	621	this work
**3c**	237 (27.3), 283 (61.4), 343 (9.65), 358 (9.55), 456 (10.4)	639	this work
**4a**	222 (32.0), 237 (31.3), 282 (47.9), 380 (6.88), 431 (9.26)	476, 622	this work
**4b**	237 (31.0), 281 (64.7), 379 (2.84), 415 (13.6)	629	this work
**4c**	238 (29.8), 283 (58.7), 338 (7.27), 375 (7.87), 420 (11.1), 445 (11.3)	640	this work
**5**	235 (31.3), 282 (62.3), 368 (7.58), 441 (11.4)	615	this work
**6**	238 (28.2), 282 (60.7), 367 (7.16), 446 (10.8)	614	this work

**Table 3:** Emission Data of Complexes in Different Solvents

	emission						
compd	solvent	10^–2^Ø	τ (ns)	χ^2^	*k*_r_ (10^4^ s^–1^)	*k*_nr_ (10^4^ s^–1^)	ref
**1a**	THF	1.4	46.1	1.01	30.6	2140	(39)
**1b**	MeCN	0.15	42.1	1.30	3.56	2370	(39)
**1c**	MeCN	0.92	178	1.18	5.15	555	(39)
**3a**	THF	4.1	50.1	1.15	82.4	1910	this work
**3b**	THF	0.45	72.0	1.16	6.25	1380	this work
**3c**	THF	6.1	304	0.99	20.0	309	this work
**4a**	MeCN	1.7	10.9	1.09	156	9020	this work
**4b**	THF	0.28	78.1	1.31	3.59	1280	this work
**4c**	MeCN	0.16	195	1.01	0.821	512	this work
**5**	H_2_O	0.50	22.9	1.01	21.8	4350	this work
**6**	H_2_O	0.41	22.8	1.03	18.0	4370	this work

The emission spectra of all the 1,2-azolylamidino complexes display one unstructured broad band in the 610–650 nm region that is solvent-dependent (shifts of ca. 20 nm for all the complexes). The intensities exhibit a severe increase in deaerated solutions in comparison to those prepared without exclusion of air, with no variation in the emission maxima (Figure S20). These results, as well as the luminescent emission lifetimes (see below), are typical of ^3^MLCT phosphorescent emissions.^65,66^

As can be observed in Table 2 and Figure 3, complex **4a** displays an unexpected behavior. In addition to the expected maximum at 613–646 nm, characteristic of Ru(II) polypyridyl complexes,^55−58^ a second emission is detected at 476 nm. This anomalous emission is also detected in THF and acetone solutions (Figure S19). In fact, the emission band at 476 nm is more intense than that at 612 nm in acetone (Figure S19). We have also observed how the intensity of this emission band at 476 nm increases, whereas that at 622 nm decreases, when a solution of **4a** in MeCN is irradiated with white light (Figure S21). First, we tried to determine whether this anomalous emission band might be related to electronic effects. The pyrazolylamidino ligand in **4a** is the result of coupling pzH and PhCN. The donor properties of the pyrazolyl moiety are between those of the more electron withdrawing indazolyl (in **4b**) and the more electron donating dimethylpyrazolyl (in **4c**). This sequence is supported by the CO stretching absorptions of *fac*-Re(CO)_3_ complexes^38,67,68^ and by the acidity of free 1,2-azoles, experimentally determined.^69,70^ Therefore, a consideration that the anomaly of this complex comes from the pyrazolyl fragment of the bidentate ligand would be difficult to accept. Thus, we turned our attention to the nitrile fragment. The PhCN used to form the 1,2-azolylamidino ligands in complexes **4** is less donating in comparison to the MeCN used to obtain complexes **3**. In order to study whether the electronic effects of the phenyl group affects the anomalous photophysical properties of **4a**, structurally similar complexes containing a donor group (methyl) or an acceptor group (F) in the para position of the phenyl group were synthesized (the NH=C(*p*-Tol)pz-κ^2^*N*,*N* ligand in **5**, and the NH=C(*p*-FC_6_H_4_)pz-κ^2^*N*,*N* ligand in **6**). However, the emission spectra of complexes **5** and **6** (Table 2 and Figure 3) are again similar to those of the complexes described herein, except for **4a**. Therefore, the electronic parameters do not provide an explanation for the anomalous emission band of **4a**. The emission spectra at 77 K (Figure S22) and in the solid state (Figure S23) were also recorded after suggestions by the reviewers, but they do not provide additional information. We have tried to carry out theoretical calculations to explain this anomaly, but unfortunately all of our attempts have failed so far. Finally, it should be pointed out that dissociation of the 1,2-azolylamidino ligand is not possible, since we have previously described how decoordination of the 1,2-azolylamidino ligands leads to reversal of the coupling reaction of the 1,2-azole and the nitrile, giving again the 1,2-azole–nitrile mixed precursor.^38^ In this case, prolonged heating at 40 °C (4 h) or irradiation (6 h) of **4a** in (CD_3_)_2_CO afforded mainly an unmodified product and only negligible amounts of the corresponding aquo and acetone complexes, *cis*-[Ru(bipy)_2_(L)(pzH)](OTf)_2_ (L = H_2_O (**2a**), (CD_3_)_2_CO). Therefore, this band cannot be due to a decomposition product.

Since the solvent does not influence the quantum yields or the luminescent emission lifetimes, these properties have been measured in different solvents, subjected to the solubilities of the complexes (Table 3). Both quantum yields and luminescent emission lifetimes are similar to those described for other ruthenium complexes.^58,71,72^ The data of dimethylpyrazolylamidino complex **3c** are remarkable, as they present the highest quantum yield and also the highest luminescence emission lifetime among all the complexes described herein. These results also agree with the higher activity attained by this complex toward the photocatalytic oxidation of thioethers, in comparison to the rest, as detailed below. The comparison among the quantum yields of pyrazolylamidino complexes leads to significant variations (0.017 and 0.041 for **4a** and **3a**, respectively, vs 0.014 for **1a**). When dimethylpyrazolyl complexes are considered, the quantum yield of **3c** (0.061) is also significantly higher than that of the dmpzH complex **1c** (0.0092). Compound **4a** shows a lower lifetime (10.9 ns) and, concomitantly, higher *k*_r_ and *k*_nr_ values (156 × 10^–4^ and 9020 × 10^–4^ s^–1^, respectively), which might point to ligand-based luminescence.

### Electrochemical Studies

The redox properties of complexes **1** have been previously described.^73,74^ However, for **1a**,**c** the reduction waves were not showed, for this reason we have reported them again. Moreover, herein we report the electrochemistry of the 1,2-azolylamidino complexes **3** and **4**.

Cyclic voltammograms of **3** and **4** in MeCN exhibit reversible Ru^II^/Ru^III^ oxidations between +1.13 and +1.25 V (vs SCE) (see Table 4 and complete data in the Supporting Information). These values are slightly lower than that found for [Ru(bipy)_3_]^2+^ (+1.29 V in MeCN)^75^ and ca. 0.3–0.5 V higher than those for the chlorido–1,2-azole complexes **1** (+0.78 to +0.83 V). This shift is in agreement with the replacement of the anionic, electron-donating chlorido ligand by the neutral, π-accepting amidino moiety. As expected, this positive variation in the potential is even higher in comparison with the potential of the *cis*-[Ru(bipy)_2_Cl_2_] complex.^73,74^

**Table 4:** Summary of Ground- and Excited-State Redox Potentials of Complexes **1**, **3**, and **4**

	redox potential, *E*_1/2_ (vs SCE)^a^				
complex	*E*_ox_ (V)	*E*_red_ (V)	*E*_0–0_ (eV)^b^	*E*_ox_* (V)^c^	*E*_red_* (V)^c^
**1a**	0.79	–1.52			
**1b**	0.83	–1.60			
**1c**	0.78	–1.62			
					
**3a**	1.20	–1.43, −1.60	2.27	–1.07	0.84
**3b**	1.19	–1.42, −1.58	2.28	–1.09	0.86
**3c**	1.25	–1.49	2.22	–0.97	0.73
					
**4a**	1.13	–1.46, −1.66			
**4b**	1.24	–1.46, −1.65			
**4c**	1.18	–1.58, −1.68			

a The electrochemical data were obtained for acetonitrile solutions; *E*_1/2_ values were referenced vs SCE, and the scan rate was 100 mV/s.

b Singlet state energy (*E*_0–0_) determined from the intersection of the normalized absorbance and emission spectra and converted into eV.

c Excited-state redox potentials estimated using the equation  or .

**Figure 4:**
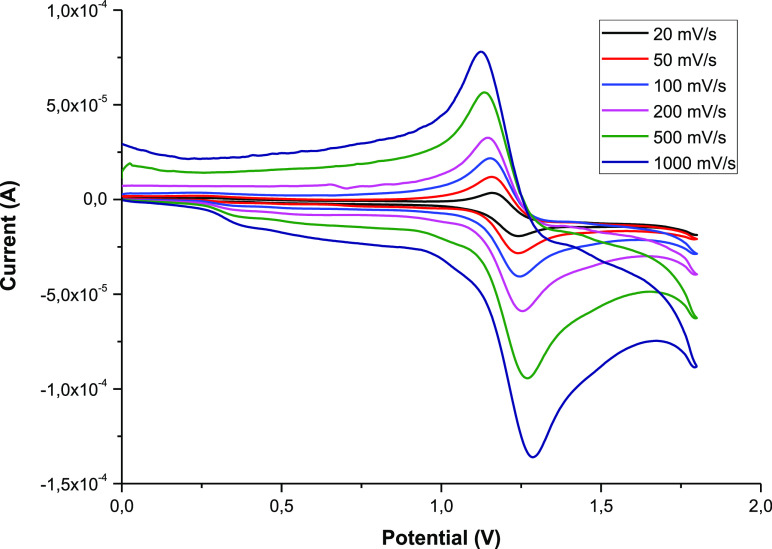
Cyclic voltammograms recorded in 2 mM acetonitrile solutions of **3a** at different scan rates (from 20 to 1000 mV/s).

Cyclic scans at different scan rates (see Figure 4 for complex **3a**) indicate that the electron transfer Ru(II) → Ru(III) is reversible.^76^ All of the 1,2-azolylamidino complexes described herein, as well as that of [Ru(bipy)_3_]^2+^, present similar behavior (see the Supporting Information).^75^

At negative potentials (0 to −1.8 V vs SCE) (see Figure S24 for **3b**), the electrochemistry of complexes **1**, **3**, and **4** is analogous to that of *cis*-bis(bipy)ruthenium(II) complexes and is associated with reduction processes centered at the bipyridine ligands.^23,73,77−79^ All of the 1,2-azolylamidino complexes described herein present similar behavior (see the Supporting Information).

From the ground-state redox potentials and the absorption and emission spectra of the Ru(II) complexes, we have estimated the excited-state redox potentials of the most catalytically active complexes **3a**–**c** (see below), which are detailed in Table 4. The excited-state oxidation and reduction potentials values of these complexes were very similar to each other, regardless of the nature of the 1,2-azolylamidino ligand of complexes **3a**–**c**.

### Photocatalytic Studies

The photocatalytic activity of the 1,2-azolylamidino complexes was tested in the oxidation of sulfides as a model reaction, using ambient oxygen as the oxidant.^26,28,80−87^ The oxidations were performed using methyl *p*-tolyl sulfide **7a** as sulfide and 1 mol % of the Ru(II) complex **3** or **4** in ethanol (Table 5). The reaction mixture was stirred open to the air under white-light irradiation. In addition, the complex [Ru(bipy)_3_]^2+^, one of the most widely used photocatalysts, was also evaluated under the same conditions. Using this complex, the oxidation of **7a** was fully accomplished in only 1 h (entry 1). With regard to the 1,2-azolylamidino complexes, the catalytic activities of **3a**–**c** (entries 2–4) were remarkably higher than those of s **4a**–**c** (entries 5–7). These results highlight the importance of the phenyl group (R′; see Scheme 2) at the 1,2-azolylamidino ligand in the activity of the photocatalyst for this transformation. In addition, the best catalytic performance was obtained with catalyst **3c**, which was able to oxidize 92% of sulfide **7a** in only 30 min (entry 4). This activity is even higher than that of [Ru(bipy)_3_]^2+^ (entry 1) under the same catalytic conditions. Finally, the catalytic performance of the precursors **1b**,**c** and **2**, as selected examples, was also evaluated (entries 8−10), these being totally inactive photocatalysts for this transformation.

**Table 5:**
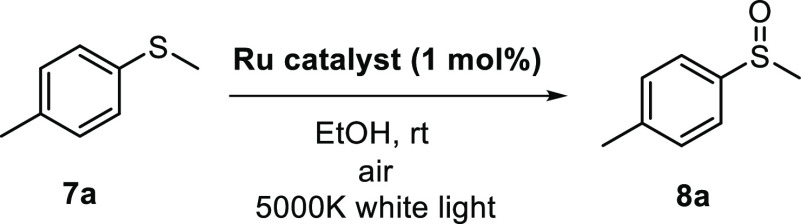
Photooxidation of Sulfide **7a** with 1,2-Azolylamidino Complexes **3** and **4**^a^

		conversion (%)^b^		
entry	catalyst	*t* = 15 min	*t* = 30 min	*t* = 60 min
1	[Ru(bipy)_3_]^2+^	60	84	100
2	**3a**	13	18	30
3	**3b**	17	40	81
4	**3c**	89	92	100
5	**4a**	1	2	10
6	**4b**	1	6	10
7	**4c**	1	2	5
8	**1b**	0	0	0
9	**1c**	0	0	0
10	**2b**	0	0	0

a Reaction conditions: open vials containing **7a** (0.2 mmol) and 1 mol % of the corresponding Ru(II) complex in 2 mL of EtOH were irradiated under white light for the indicated time.

b Determined by ^1^H NMR analysis of the crude mixture.

The catalytic performance should be related to the physical properties of the catalyst. Therefore, the absorption and emission spectra, quantum yields, and lifetimes in MeOH and CD_3_OD were measured for **3c**, which showed the best catalytic performance. The results are collected in Figure S26 and Table S10 in the the Supporting Information. The spectra of **3c** in MeOH and CD_3_OD are very similar to those in MeCN, THF, and acetone (Figure S19), although the lifetime is reduced in methanol (131 ns for MeOH, 136 ns for CD_3_OD) in comparison to THF (304 ns in THF). There are no significant differences between the data in MeOH and those in CD_3_OD.

**Scheme 3:**
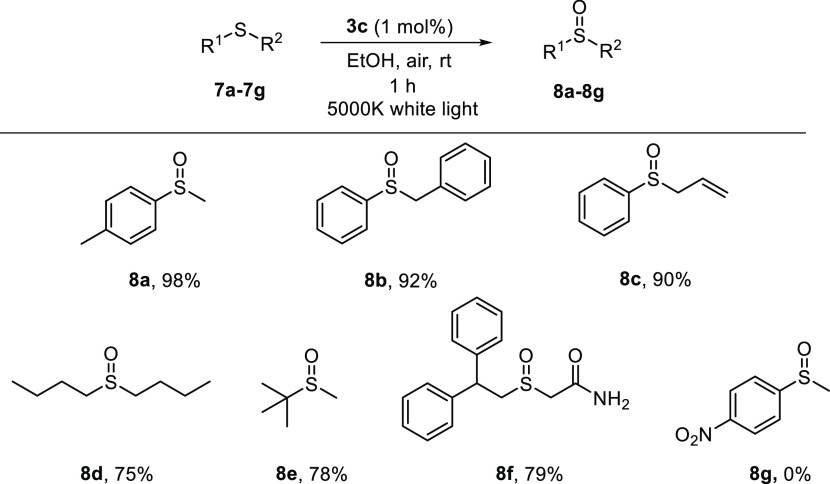
Scope of the Photooxidation Reaction of Sulfides **7** using Catalyst **3c**

Next, the activity of catalyst **3c** was studied using sulfides of different nature under the best reaction conditions (Scheme 3). Complex **3c** was able to chemoselectively catalyze the oxidation of benzyl sulfide **7b** and allyl sulfide **7c** in high yields, without detection of any other byproduct from the oxidation of the benzylic position or the doble bond. Dialkyl sulfides **7d**,**e** were also easily oxidized, the corresponding sulfoxides being isolated in 75% and 78% yields, respectively. Conversely, the oxidation of methyl *p*-nitrophenyl sulfide (**7g**) did not take place, suggesting that the oxidation reaction goes through a photoredox mechanism, as explained below. Finally, the applicability of the catalytic system was evaluated in the preparation of the drug modafinil, which is a wake-promoting agent.^88^ This drug (**8f**) was also successfully prepared in 79% yield in only 1 h using complex **3c** as the photocatalyst, under environmentally friendly oxidation conditions. It is important to mention that the oxidation proceeds chemoselectively to the formation of the sulfoxide in all of the cases studied, overoxidation to the sulfone never being observed.

**Figure 5:**
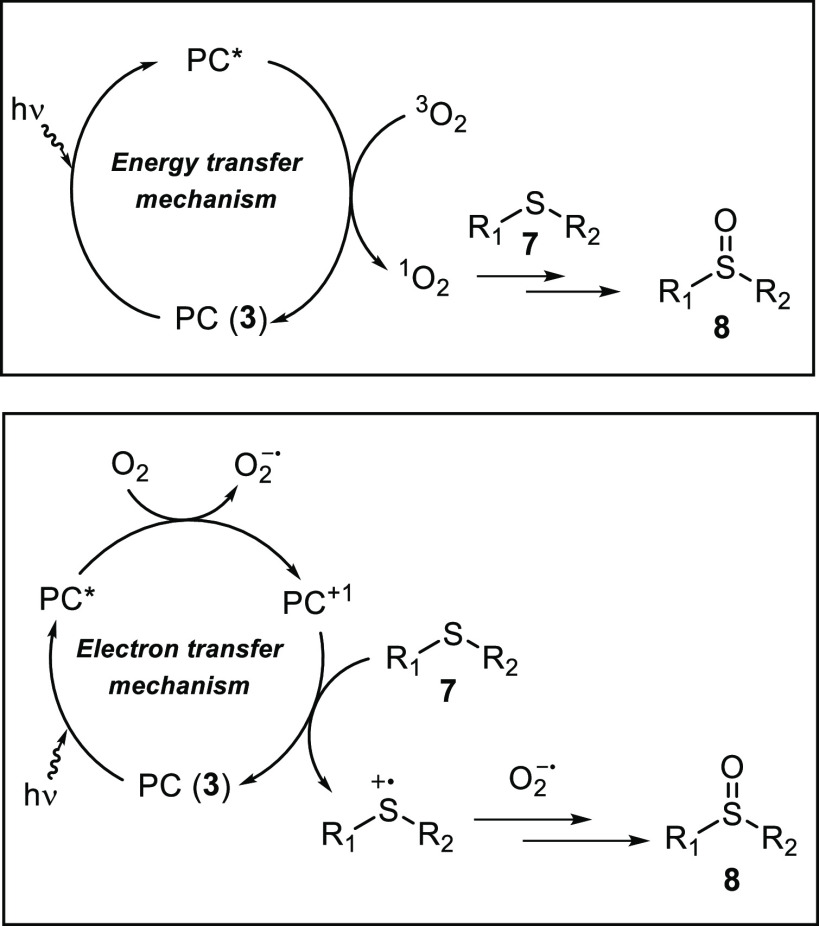
Two possible mechanistic pathways for the photooxidation of sulfides under visible-light irradiation.

With regard to the mechanism, two different pathways are possible for the photoxidation of sulfides (Figure 5): (a) an energy transfer process, which produces singlet oxygen, or (b) a photoredox process, which involves superoxide radical anion and sulfide radical cation species.^81^ Distinguishing between both mechanisms is not easy, but the addition of selective quenchers or enhancers of the reactive oxygen species can help to identify the predominant pathway. These experiments were performed with methyl *p*-tolyl sulfide (**7a**) in MeOH as solvent, and the reaction was stopped after 10 min (Table 6). Parallel experiments were carried out using catalyst **3c** or [Ru(bipy)_3_]^2+^, and analogous results were obtained in all of the experiments. It is known that the use of deuterated solvents accelerates oxidation reactions mediated by singlet oxygen.^82^ In our case, similar results were obtained when the reactions were performed using MeOH or CD_3_OD (entries 1 and 2). Conversely, the addition of sodium azide as a scavenger of the singlet oxygen species completely inhibited the reaction (entry 3), pointing to an energy transfer process. On the other hand, the outcome of the oxidation of **7a** in the presence of a scavenger of the sulfide radical cation, such as 1,4-dimethoxybenzene, or a scavenger of the superoxide radical, such as benzoquinone, would indicate the contribution of the photoredox pathway.^81^ Moreover, while the presence of benzoquinone fully suppressed the oxidation process (entry 5), the addition of 1,4-dimethoxybenzene induced a slight decrease in the formation of **8a** (entry 4). An analogous mechanistic study with scavengers was carried out using catalysts **3a**,**b**, giving the same inhibition trends (Table S11). All of the mechanistic experiments, together with the unsuccessful oxidation of the *p*-nitro sulfide derivative **7f**, suggest the participation of radical intermediates in the mechanism. In summary, all of these mechanistic experiments point out that both energy transfer and photoredox processes are taking place in the photooxidation of sulfides using the 1,2-azoylamidino ruthenium complexes **3** as catalysts.

**Table 6:**
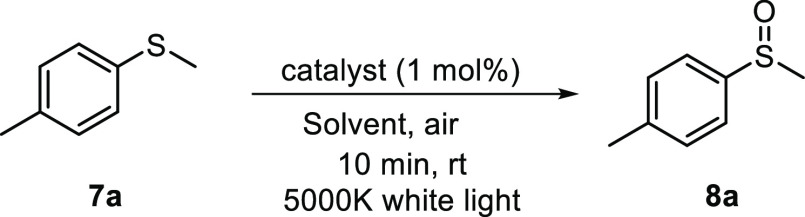
Mechanistic Tests Using Scavengers or Enhancers

				conversion (%)^a^	
entry	solvent	additive (0.5 equiv)	aim	**3c**	[Ru(bipy)_3_]^2+^
1	CH_3_OH			60	58
2	CD_3_OD		enhancing ^1^O_2_-mediated pathway	65	62
3	CH_3_OH	NaN_3_	^1^O_2_ scavenger	0	0
4	CH_3_OH	1,4-dimethoxybenzene	R_2_S^+^ scavenger	53	57
5	CH_3_OH	benzoquinone	O_2_^–^ scavenger	0	0

a Conversion determined by an ^1^H NMR analysis of the crude mixture.

## Conclusions

A new family of *cis*-Ru^II^(bipy)_2_ complexes with the 1,2-azolylamidino ligand has been synthesized and thoroughly characterized by ^1^H, ^13^C, and ^15^N NMR and IR spectroscopy and single-crystal X-ray diffractometry. The complexes show phosphorescent emissions with quantum yields and lifetimes comparable to those of other analogous complexes. The redox properties are similar to those of [Ru(bipy)_3_]^2+^, with reversible Ru^II^/Ru^III^ oxidations between +1.13 and +1.25 V (vs SCE). Moreover, the 1,2-azolylamidino complexes can be used as catalysts in the photooxidation of different thioethers. In fact, the dimethylpyrazolylamidino complex *cis*-[Ru(bipy)_2_(NH=C(Me)dmpz-κ^2^*N*,*N*)]^2+^, which presents the highest quantum yield and also the highest luminescent emission lifetime, shows a better catalytic performance in comparison to that of [Ru(bipy)_3_]^2+^. Among the wide range of *cis*-Ru^II^(bipy)_2_ complexes reported, the family of 1,2-azolylamidino complexes described herein has the advantages of facile synthesis and the ability to fine-tune the electrochemical, luminescent, and catalytic activity by varying the steric and electronic effects of the 1,2-azole and nitrile precursors.

## Supporting Information Available

Additional characterization and experimental data and crystal structures of complexes **3a**–**c** and **4a**–**c** (PDF)

## Accession Codes

CCDC 2044577–2044582 contain the supplementary crystallographic data for this paper. These data can be obtained free of charge via www.ccdc.cam.ac.uk/data_request/cif, or by emailing data_request@ccdc.cam.ac.uk, or by contacting The Cambridge Crystallographic Data Centre, 12 Union Road, Cambridge CB2 1EZ, UK; fax: +44 1223 336033.
