# Supplementary material for: Luminescent cis-Bis(bipyridyl)ruthenium(II) Complexes with 1,2-Azolylamidino Ligands: Photophysical, Electrochemical Studies, and Photocatalytic Oxidation of Thioethers
Source: Inorg Chem. 2021 Apr 27;60(10):7008–22. doi: 10.1021/acs.inorgchem.0c03389 (PMC8812113; doi:10.1021/acs.inorgchem.0c03389)
Supplement: Supplementary file 1 — ic0c03389_si_001.pdf [file ic0c03389_si_001.pdf]

SUPPLEMENTARY INFORMATION

Luminescent *cis*-bis(bipyridyl)ruthenium(II) complexes with 1,2-azolylamidino ligands: photophysical, electrochemical studies, and photocatalytic oxidation of thioethers

Elena Cuéllar,[a] Alberto Diez-Varga,[b] Tomás Torroba,[b] Pablo Domingo-Legarda,[c] José Alemán,[c] Silvia Cabrera,[c] Jose M. Martín-Alvarez,[a] Daniel Miguel,[a] and Fernando Villafañe[a,*]

[a] GIR MIOMeT-IU Cinquima-Química Inorgánica, Facultad de Ciencias, Campus Miguel Delibes, Universidad de Valladolid, 47011 Valladolid, Spain.

[b] Departamento de Química, Facultad de Ciencias, Universidad de Burgos, 09001 Burgos, Spain.

[c] Departamento de Química Inorgánica, Facultad de Ciencias, Universidad Autónoma de Madrid, 28049 Madrid, Spain.

Index

^{1}H and ^{13}C NMR spectra 4

Figure S1. ^{1}H NMR spectra of 3a at 298 K....4

Figure S2. ^{13}C NMR spectra of 3a at 298 K....4

Figure S3. ^{1}H NMR spectra of 3b at 298 K. 4

Figure S4. ^{13}C NMR spectra of 3b at 298 K. 5

Figure S5. ^{1}H NMR spectra of 3c at 298 K....5

Figure S6. ^{13}C NMR spectra of 3c at 298 K....5

Figure S7. ^{1}H NMR spectra of 4a at 298 K....6

Figure S8. ^{13}C NMR spectra of 4a at 298 K....6

Figure S9. ^{1}H NMR spectra of 4b at 298 K. 6

Figure S10. ^{13}C NMR spectra of 4b at 298 K. 7

Figure S11. ^{1}H NMR spectra of 4b at 243 K. 7

Figure S12. ^{13}C NMR spectra of 4b at 243 K. 7

Figure S13. ^{1}H NMR spectra of 4c at 298 K....8

Figure S14. ^{13}C NMR spectra of 4c at 298 K....8

Figure S15. ^{1}H NMR spectra of 4c at 243 K....8

Figure S16. ^{13}C NMR spectra of 4c at 243 K....9

Figure S17. ^{1}H NMR spectra of 4c at 298 K (above) and 243 K (below)....10

Figure S18. ^{13}C NMR spectra of 4c at 298 K (above) and 243 K (below)....10

Photophysical studies 11

Figure S19. Normalized UV/vis absorption (black) and emission (blue, λ_{ex} = 420 nm) spectra at 298 K, in deaerated solvents in optically dilute solutions for (a) 3a, (b) 3b, (c) 3c, (d) 4a, (e) 4b, (f) 4c, (g) 5, and (h) 6 (above). Absorption and emission (emission intensity decrease = 100 · $I_{non-deaerated}/I_{aerated}$) data at 298 K, in different solvents (below). 14

Figure S20. Emission spectra of 4c in aerated (black) and deaerated (red) MeCN. 15

Figure S21. Evolution of the emission spectra of 4a in MeCN over time. Initial (black) and after irradiation under white light for 24 hours (red). 15

Figure S22. Emission spectra of 4a in MeCN at 278 K (black) and at 77K (red). 15

Figure S23. Emission spectra of 4a in the solid state. 16

Cyclic voltammograms Figures and Tables 17

Figure S24. Cyclic voltammogram recorded in 2 mM acetonitrile solutions of 3b at 20 mV/s. 17

Table S1. $[Ru(bpy)_2Cl(pzH)]OTf$, 1a 18

Table S2. $[Ru(bpy)_2Cl(IndzH)]OTf$, 1b 19

Table S3. $[Ru(bpy)_2Cl(dmpzH)]OTf$, 1c 20

Table S4. [Ru(bpy)$_2$(NH=C(Me)pz-κ^{2}N,N)](OTf)$_2$, 3a 21
Table S5. [Ru(bpy)$_2$(NH=C(Me)IndzH-κ^{2}N,N)](OTf)$_2$, 3b 22
Table S6. [Ru(bpy)$_2$(NH=C(Me)dmpzH-κ^{2}N,N)](OTf)$_2$, 3c 23
Table S7. [Ru(bpy)$_2$(NH=C(Ph)pz-κ^{2}N,N)](OTf)$_2$, 4a 24
Table S8. [Ru(bpy)$_2$(NH=C(Ph)Indz-κ^{2}N,N)](OTf)$_2$, 4b 25
Table S9. [Ru(bpy)$_2$(NH=C(Ph)dmpz-κ^{2}N,N)](OTf)$_2$, 4c..26
Light system for performing the photocatalytic reactions 27
Figure S25. Custom-made temperature-controlled photocatalytic system (left). Emission spectrum of the 5000 K White LED (right). The picture has been taken by the authors of the manuscript. 27
Figure S26. Normalized UV/vis absorption (black) and emission (blue, λ_{ex} = 420 nm) spectra of 3c at 298 K, in deaerated MeOH (left) and CD_3OD (right) in optically dilute solutions.............................27
Table S10. Quantum yields and lifetimes of 3c in MeOH and CD_3OD. 27
Table S11. Mechanistic tests using scavengers. 28

^{1}H and ^{13}C NMR spectra

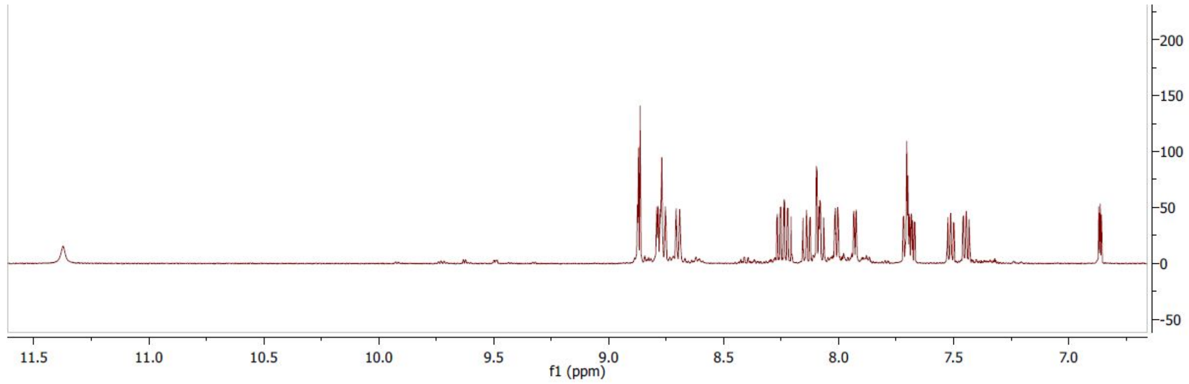

Figure S1. ^{1}H NMR spectra of 3a at 298 K.

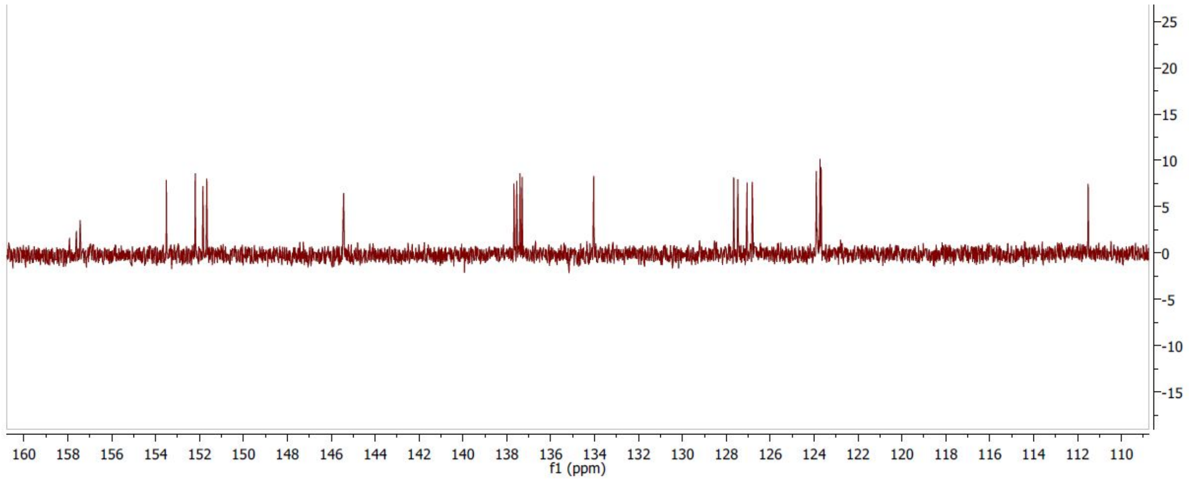

Figure S2. ^{13}C NMR spectra of 3a at 298 K.

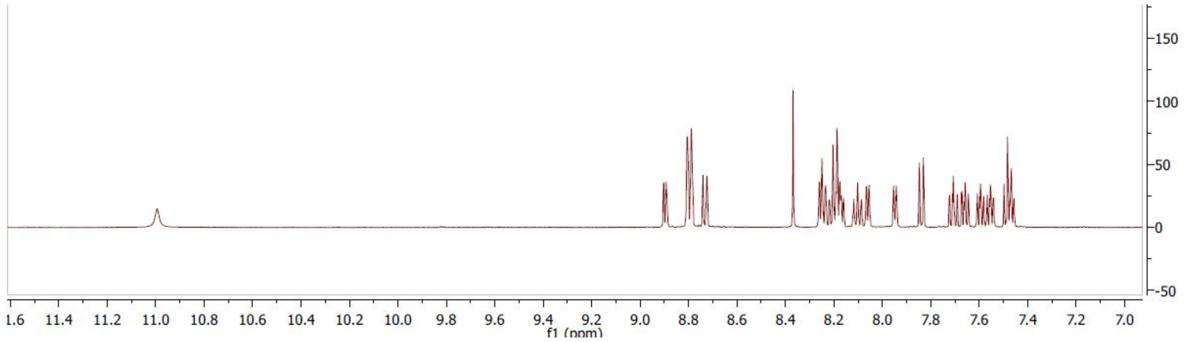

Figure S3. ^{1}H NMR spectra of 3b at 298 K.

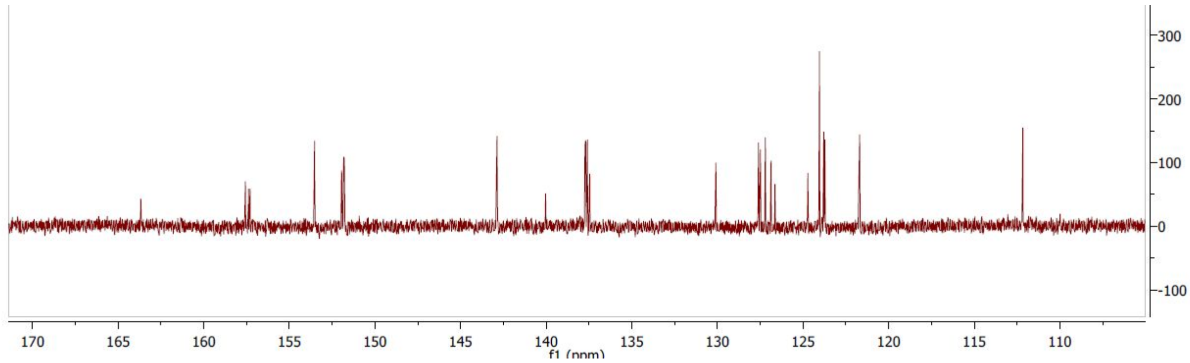

Figure S4. ^{13}C NMR spectra of 3b at 298 K.

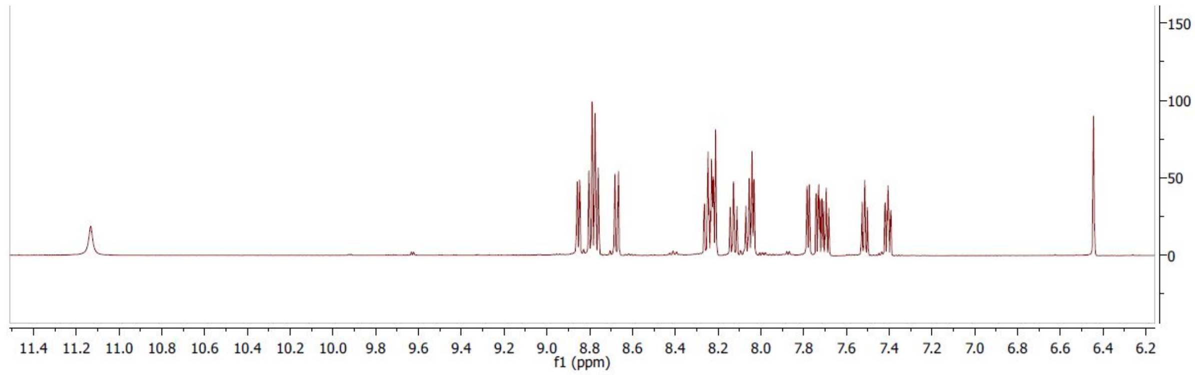

Figure S5. ^{1}H NMR spectra of 3c at 298 K.

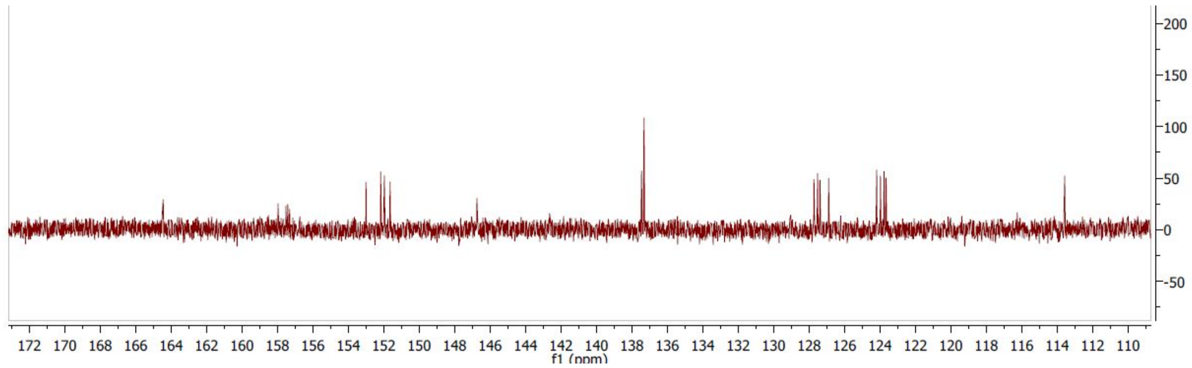

Figure S6. ^{13}C NMR spectra of 3c at 298 K.

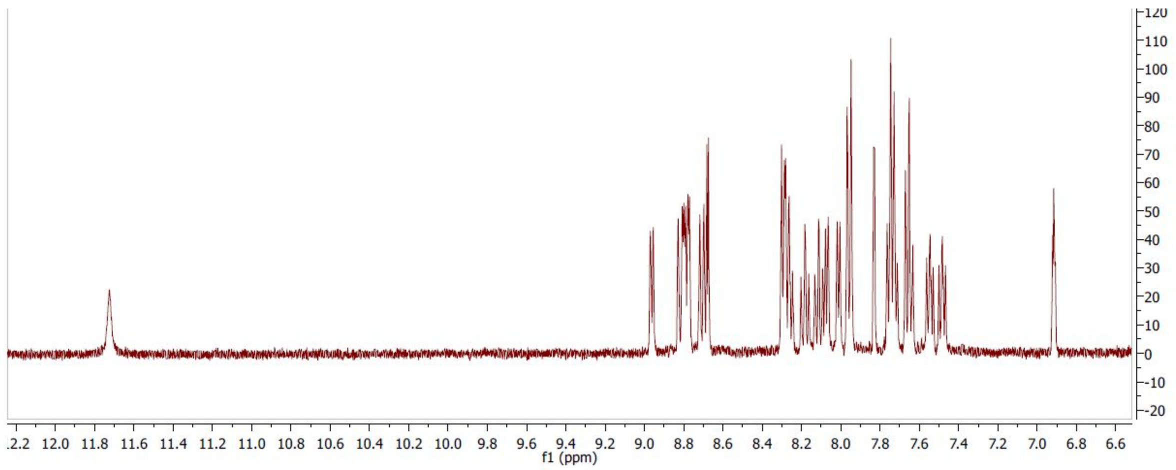

Figure S7. ^{1}H NMR spectra of 4a at 298 K.

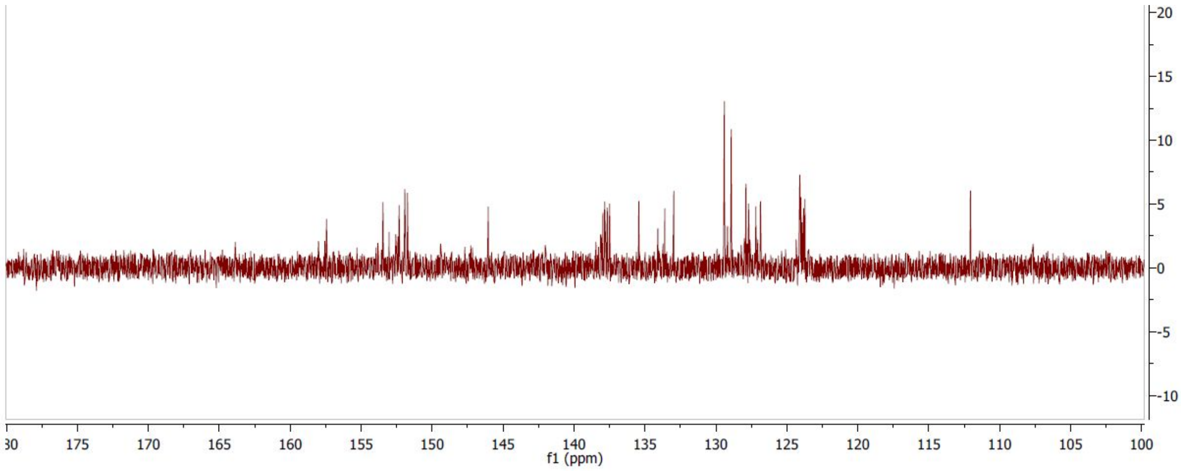

Figure S8. ^{13}C NMR spectra of 4a at 298 K.

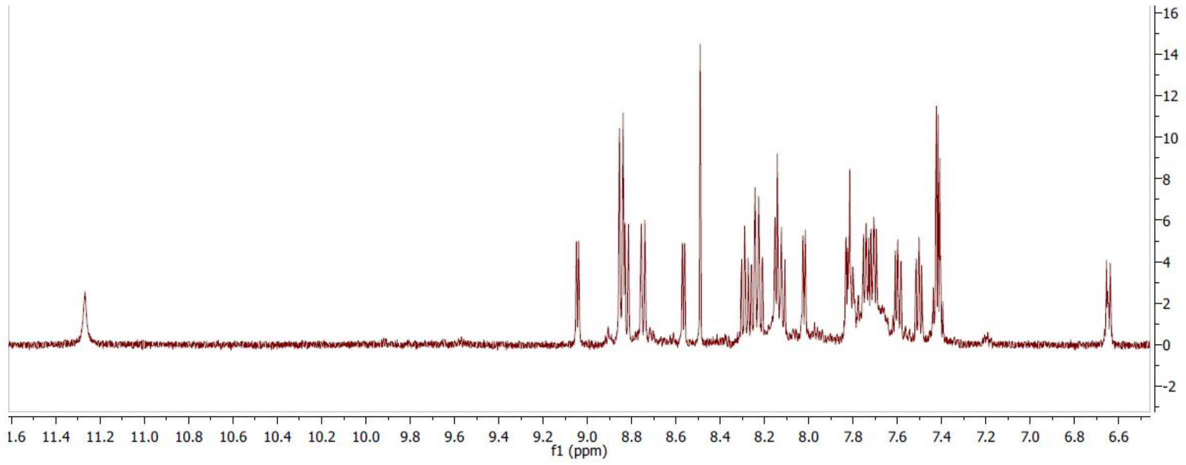

Figure S9. ^{1}H NMR spectra of 4b at 298 K.

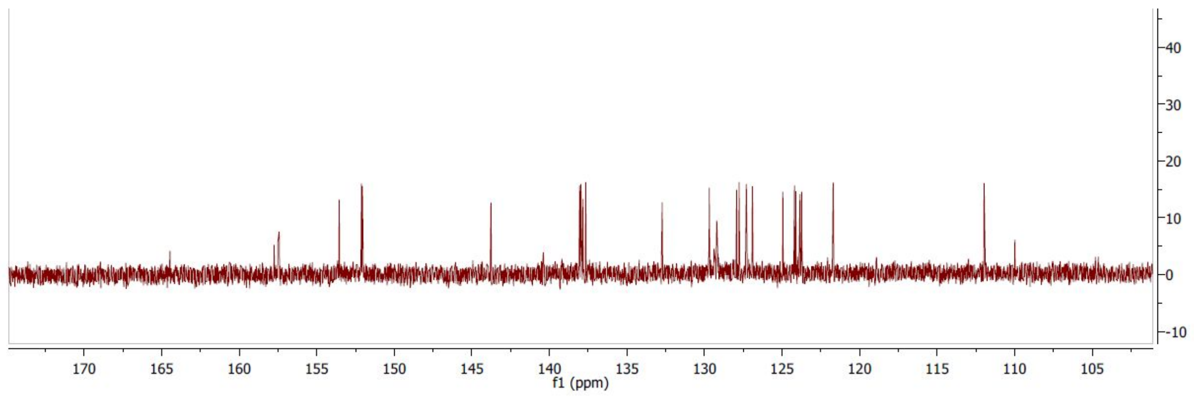

Figure S10. ^{13}**C NMR spectra of 4b** at 298 K.

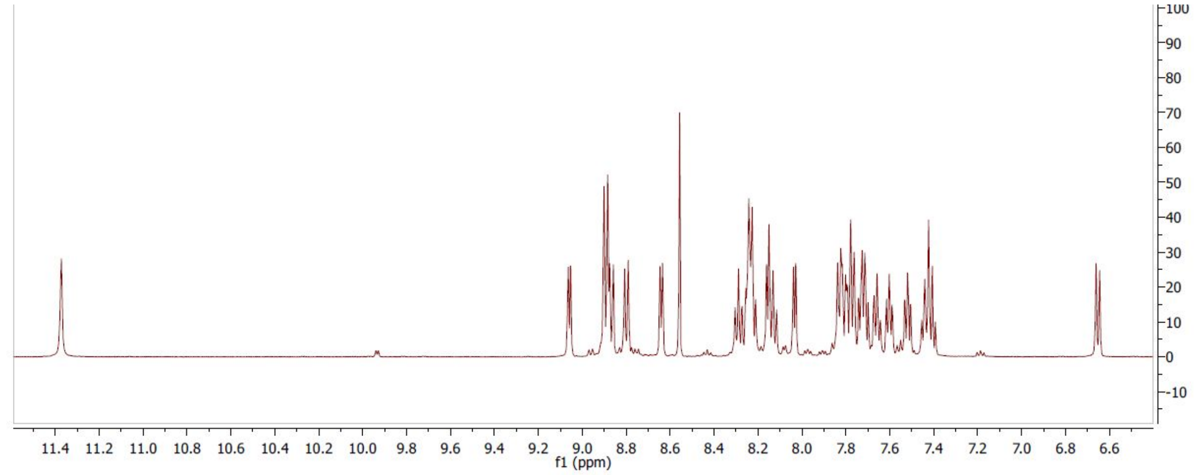

Figure S11. ^{1}**H NMR spectra of 4b** at 243 K.

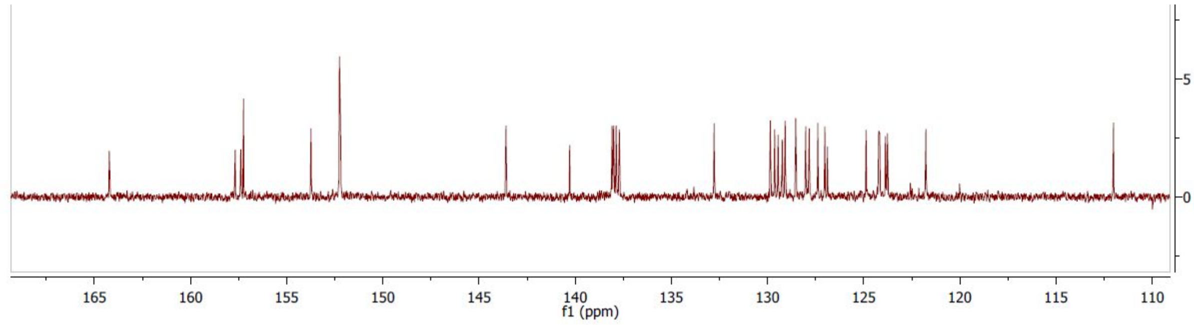

Figure S12. ^{13}**C NMR spectra of 4b** at 243 K.

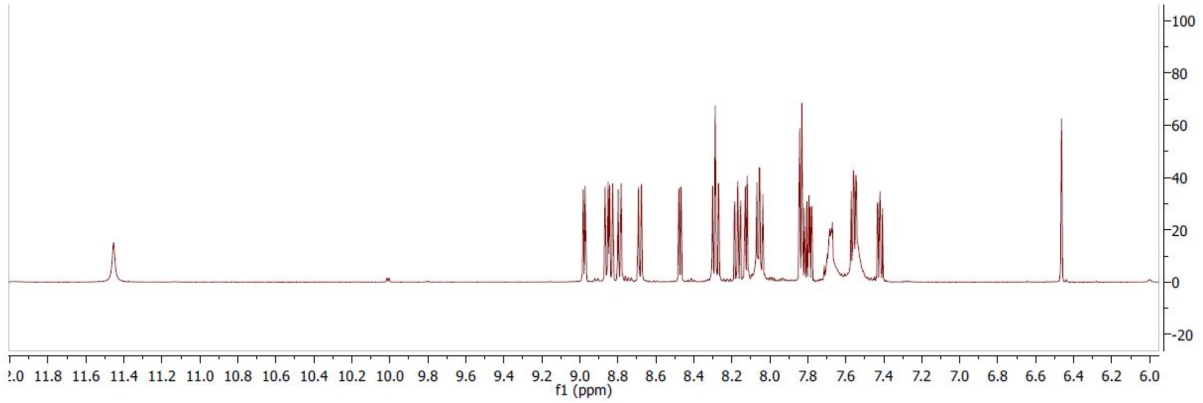

Figure S13. ^{1}H NMR spectra of 4c at 298 K.

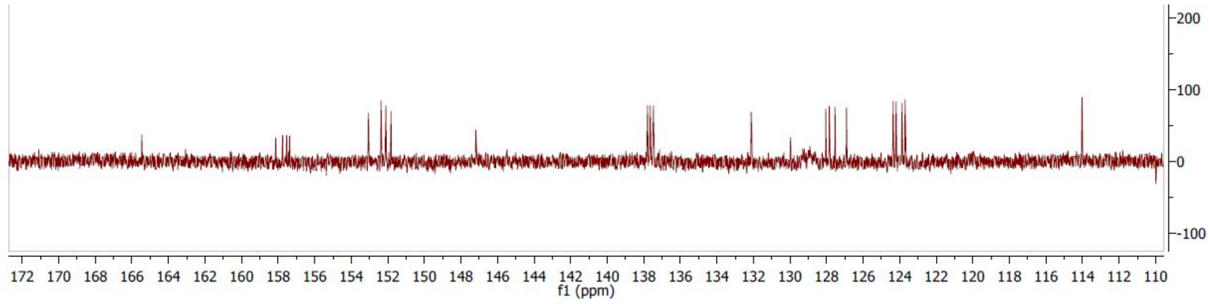

Figure S14. ^{13}C NMR spectra of 4c at 298 K.

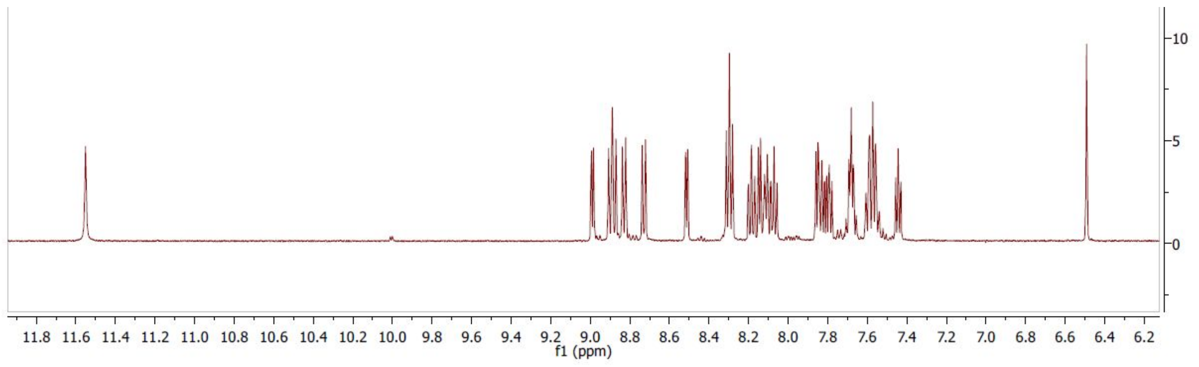

Figure S15. ^{1}H NMR spectra of 4c at 243 K.

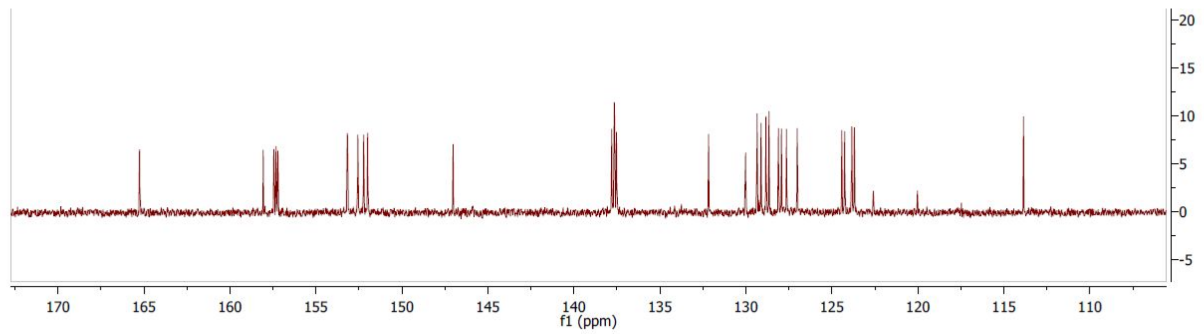

Figure S16. ^{13}C NMR spectra of 4c at 243 K.

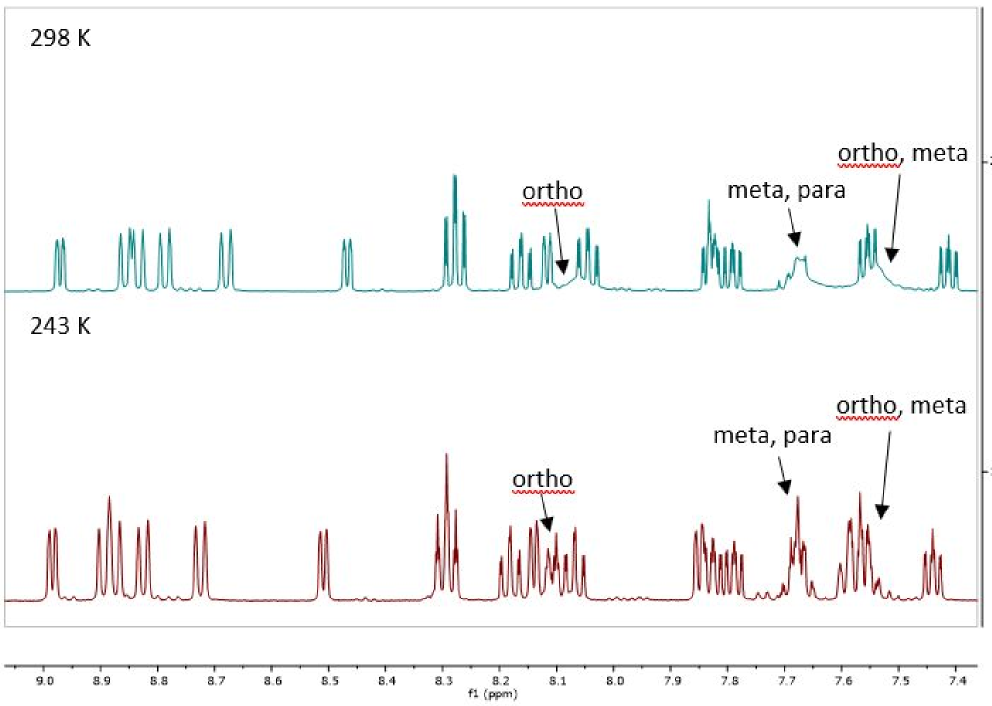

Figure S17. ^{1}H NMR spectra of 4c at 298 K (above) and 243 K (below).

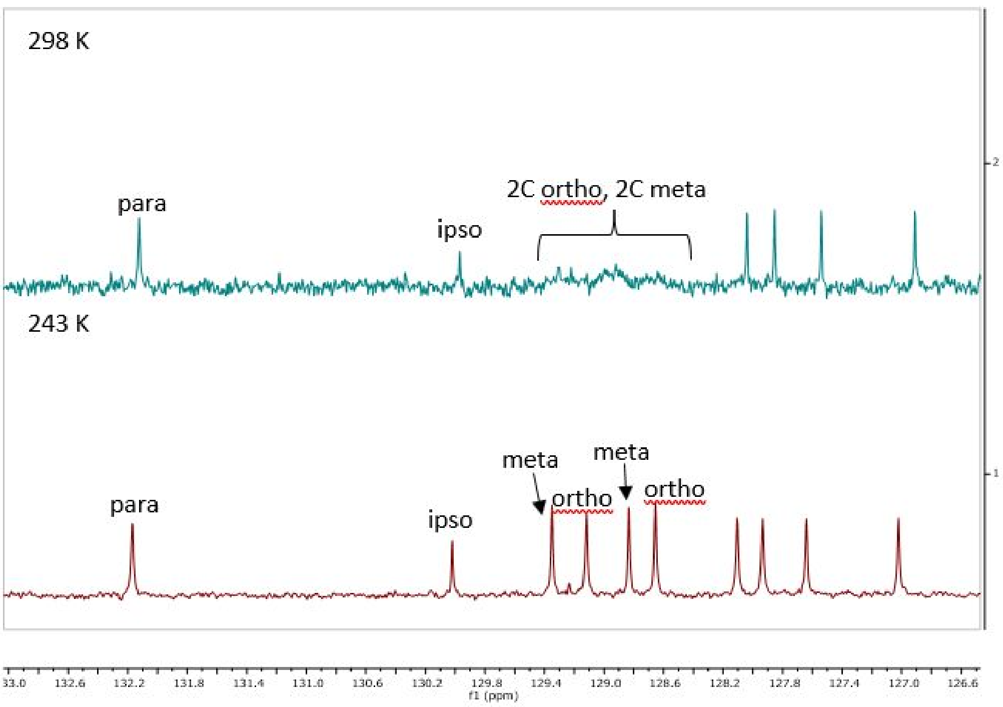

Figure S18. ^{13}C NMR spectra of 4c at 298 K (above) and 243 K (below).

Photophysical studies

a)

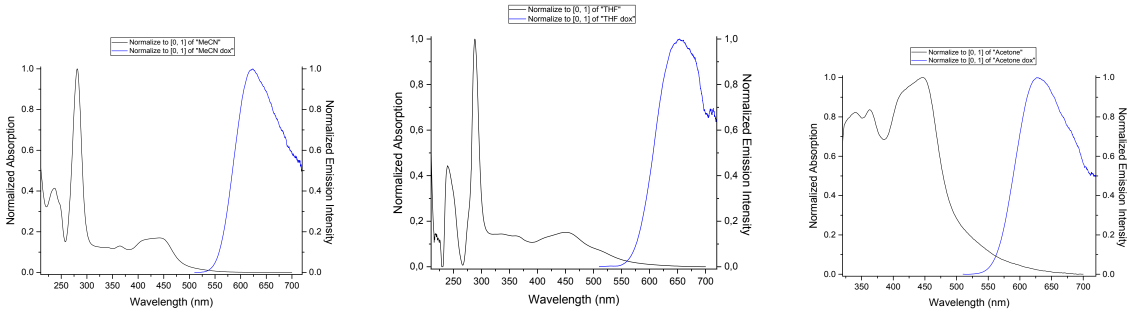

3a	Absorption λ nm (ε $M^{-1}cm^{-1}$)	Emission λ nm (% decrease)
MeCN	237 (23300), 281 (56400), 365 (7270), 442 (9510)	622 (14%)
THF[a]	240 (14700), 288 (33000), 327 (4740), 361 (4500), 451 (5010)	652 (24%)
Acetone	341 (8080), 363 (8200), 448 (9800)	629 (23%)

[a] The solubility of **3a** in THF is low

b)

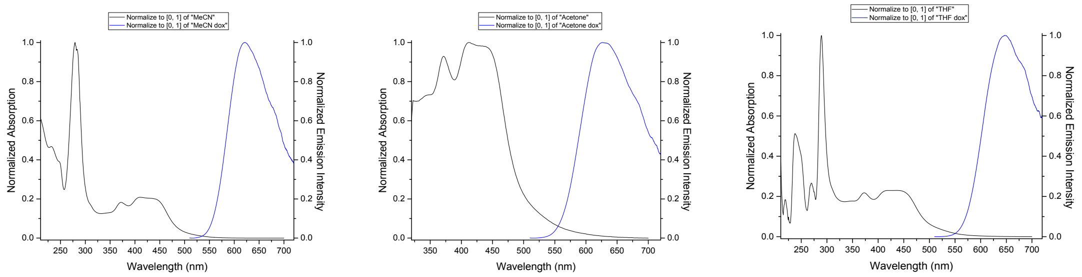

3b	Absorption λ nm (ε $M^{-1}cm^{-1}$)	Emission λ nm (% decrease)
MeCN	232 (40100), 280 (85800), 373 (15600), 410 (17800)	621 (15%)
THF	238 (35900), 270 (18700), 290 (70100), 373 (15300), 421 (16100), 438 (16100)	647 (27%)
Acetone	323 (12100), 372 (16000), 412 (17300), 429 (17000), 434 (17000)	627 (33%)

c)

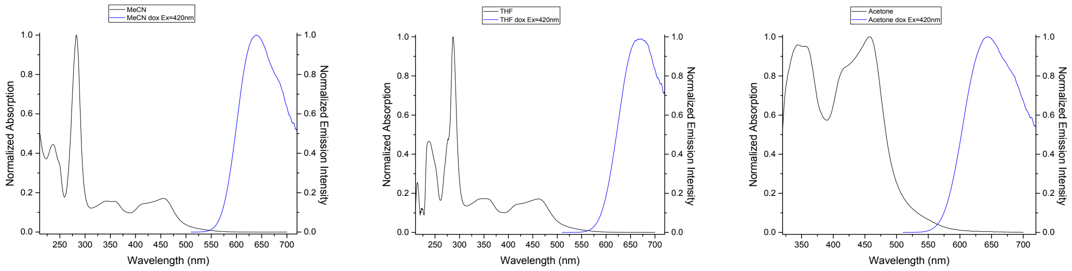

**3c**	**Absorption** λ nm (ε $M^{-1}cm^{-1}$)	**Emission** λ nm (% decrease)
MeCN	237 (27300), 283 (61400), 343 (9650), 358 (9550), 456 (10400)	639 (51%)
THF	214 (12500), 238 (22800), 287 (49000), 347 (8420), 462 (8350)	669 (58%)
Acetone	344 (10600), 356 (10500), 458 (11000)	645 (52%)

d)

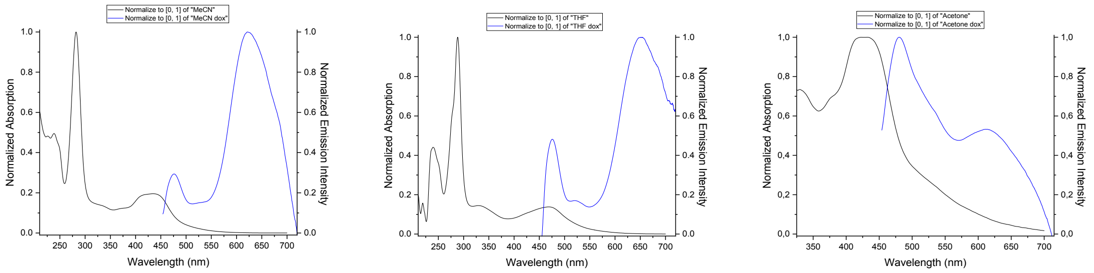

**4a**	**Absorption** λ nm (ε $M^{-1}cm^{-1}$)	**Emission** λ nm (% decrease)
MeCN	222 (32000), 237 (31300), 282 (47900), 380 (6890), 431 (9260)	476 (34%), 622 (36%)
THF	236 (30800), 287 (44400), 382 (5890), 436 (7450)	476 (28%), 653 (25%)
Acetone	330 (8630), 383 (7210), 432 (9600)	480 (47%), 612 (43%)

e)

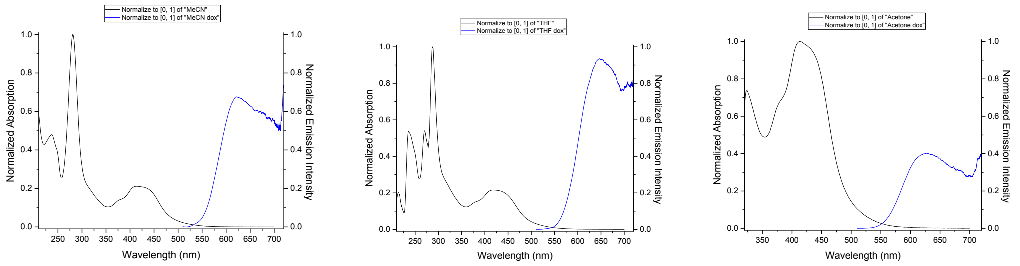

4b	Absorption λ nm (ε $M^{-1}cm^{-1}$)	Emission λ nm (% decrease)
MeCN	237 (31000), 281 (64700), 379 (2840), 415 (13600)	629 (39%)
THF	236 (30600), 270 (30900), 288 (57100), 380 (3020), 419 (12300)	649 (35%)
Acetone	324 (10900), 379 (13400), 414 (14800)	627 (28%)

f)

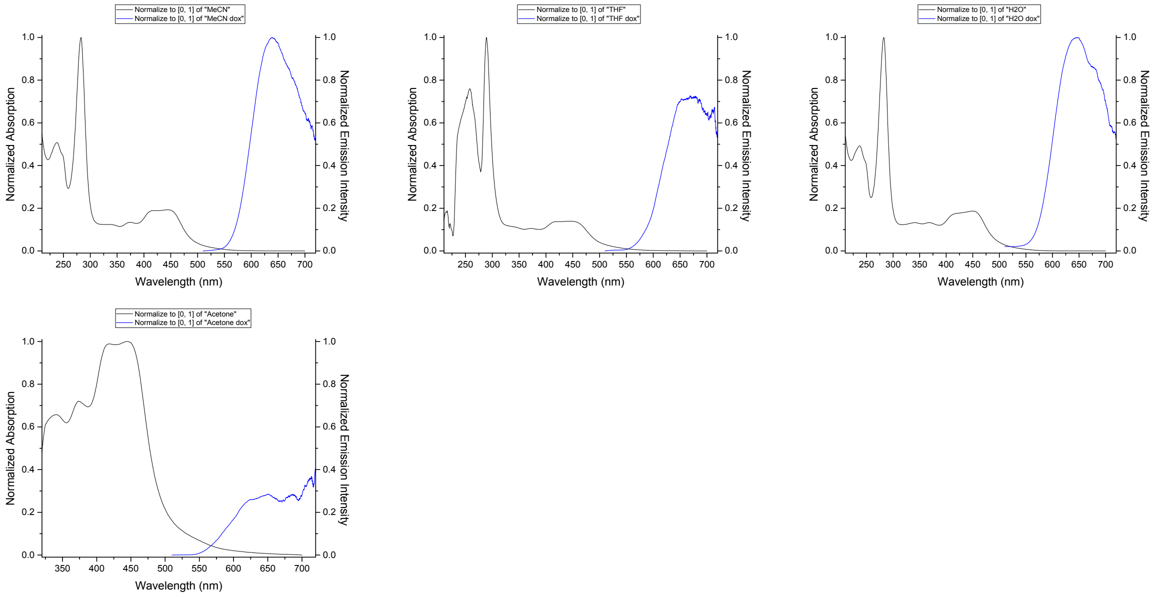

4c	Absorption λ nm (ε $M^{-1}cm^{-1}$)	Emission λ nm (% decrease)
MeCN	238 (29800), 283 (58700), 338 (7270), 375 (7870), 420 (11100), 445 (11300)	640 (59%)
THF	258 (53000), 289 (69700), 372 (7340), 421 (9560), 447 (9710)	669 (31%)
H_2O	237 (24300), 283 (49400), 342 (6570), 368 (6580), 449 (9250)	644 (20%)
Acetone	341 (7460), 374 (8170), 419 (11200), 444 (11300)	642 (45%)

g)

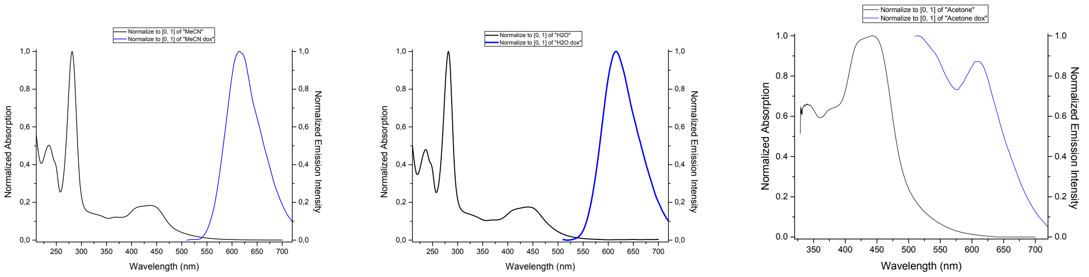

**5**	**Absorption** λ nm (ε M^{-1}cm^{-1})	**Emission** λ nm (% decrease)
MeCN	235 (31300), 282 (62300), 368 (7580), 441 (11400)	615 (1%)
H_2O	236 (28900), 282 (60200), 336 (6420), 442 (10600)	616 (1%)
Acetone	342 (10300), 443 (15700)	609 (8%)

h)

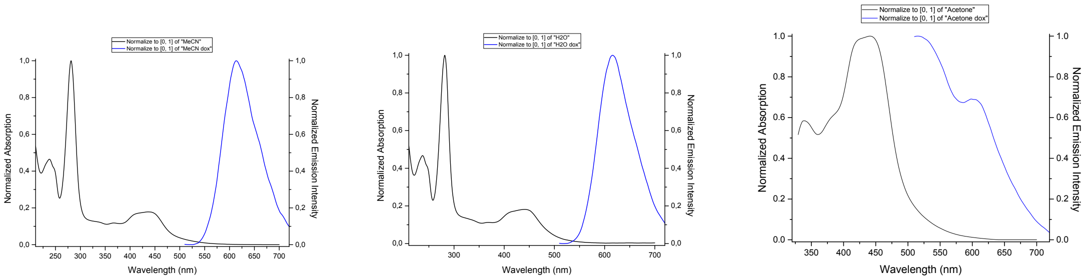

**6**	**Absorption** λ nm (ε M^{-1}cm^{-1})	**Emission** λ nm (% decrease)
MeCN	238 (28200), 282 (60700), 367 (7160), 446 (10800)	614 (7%)
H_2O	238 (27400), 282 (58700), 369 (6600), 440 (10700)	616 (4%)
Acetone	340 (10400), 441 (17800)	599 (1%)

Figure S19. Normalized UV/vis absorption (black) and emission (blue, λ_{ex} = 420 nm) spectra at 298 K, in deaerated solvents in optically dilute solutions for (a) **3a**, (b) **3b**, (c) **3c**, (d) **4a**, (e) **4b**, (f) **4c**, (g) **5**, and (h) **6** (above). Absorption and emission (emission intensity decrease = 100 · $I_{non\text{-}deaerated}/I_{aerated}$) data at 298 K, in different solvents (below).

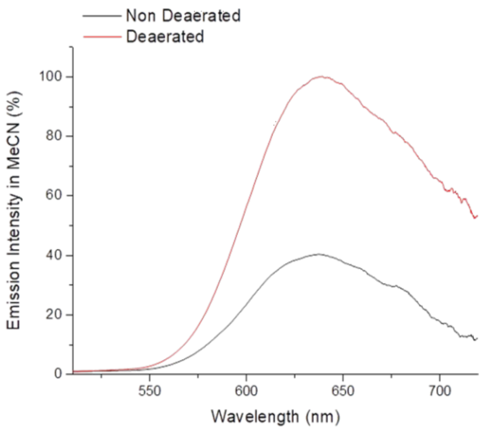

Figure S20. Emission spectra of 4c in aerated (black) and deaerated (red) MeCN.

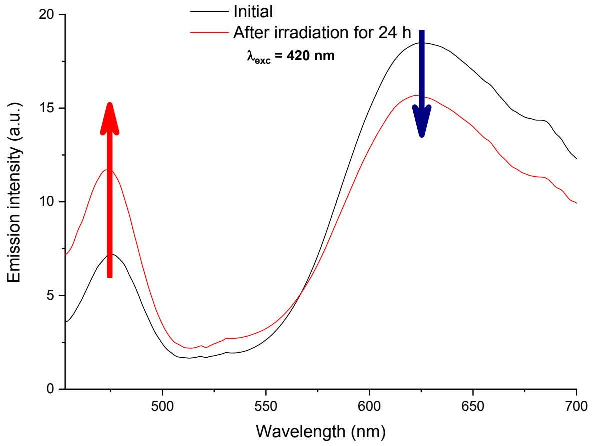

Figure S21. Evolution of the emission spectra of 4a in MeCN over time. Initial (black) and after irradiation under white light for 24 hours (red).

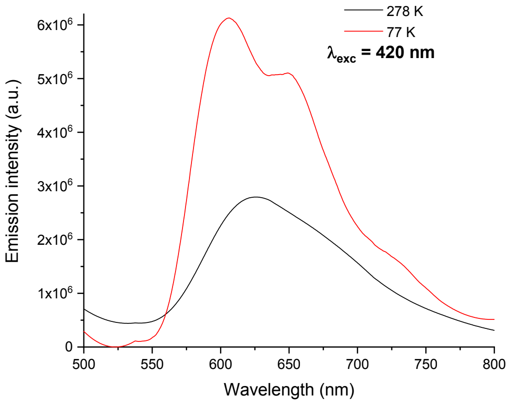

Figure S22. Emission spectra of 4a in MeCN at 278 K (black) and at 77K (red).

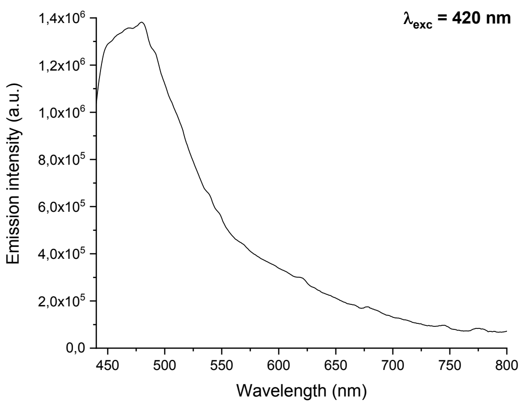

Figure S23. Emission spectra of 4a in the solid state.

Cyclic voltammograms Figures and Tables

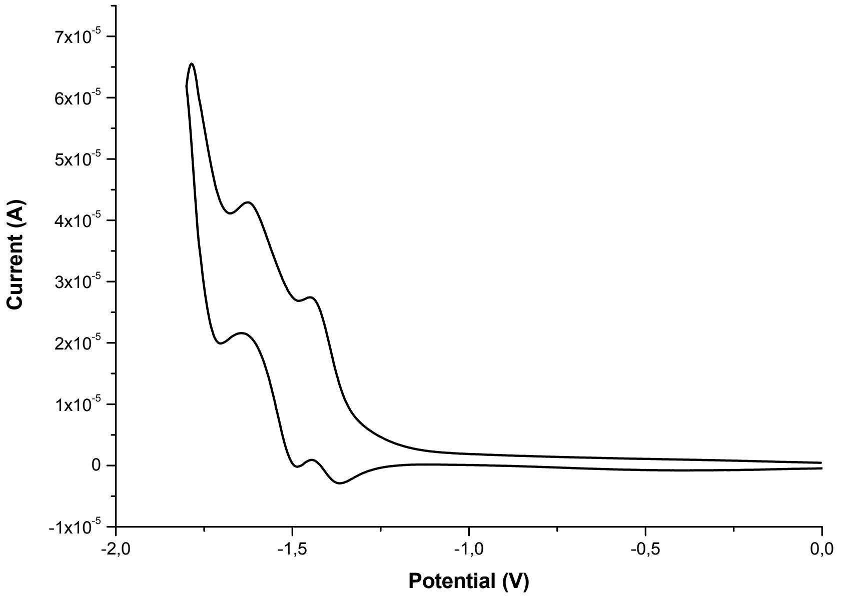

Figure S24. Cyclic voltammogram recorded in 2 mM acetonitrile solutions of 3b at 20 mV/s.

Table S1. $[Ru(bpy)_2Cl(pzH)]OTf$, **1a**

V(x) max. and min. (V)	Intensity (μA)
20 mV/s	
0.739<br>0.838	9.85<br>-24.6
-1.53<br>-1.50	26.8<br>-5.27
50 mV/s	
0.732<br>0.845	23.6<br>-40.0
-1.57<br>-1.49	45.1<br>-17.1
100 mV/s	
0.683<br>0.895	47.0<br>-53.3
-1.58<br>-1.45	64.1<br>-28.1
200 mV/s	
0.658<br>0.919	62.5<br>-68.4
-1.60<br>-1.42	88.0<br>-45.6
500 mV/s	
0.616<br>0.969	87.2<br>-100.0
-1.63<br>-1.42	139.0<br>-76.9
1000 mV/s	
0.595<br>1.01	112.0<br>-134.0
-1.66<br>-1.41	194.0<br>-108.0

Table S2. [Ru(bpy)$_2$Cl(IndzH)]OTf, **1b**

V(x) max. and min. (V)	Intensity (μA)
20 mV/s	
0.789<br>0.856	7.55<br>-19.6
-1.52<br>-1.48	19.5<br>2.57
50 mV/s	
0.778<br>0.866	17.4<br>-31.2
-1.55<br>-1.48	36.7<br>-10.9
100 mV/s	
0.775<br>0.866	31.0<br>-46.0
-1.55<br>-1.46	55.4<br>-25.9
200 mV/s	
0.760<br>0.880	45.0<br>-64.8
-1.57<br>-1.45	77.0<br>-45.5
500 mV/s	
0.760<br>0.880	45.0<br>-66.0
-1.58<br>-1.43	123.0<br>-76.8
1000 mV/s	
0.733<br>0.911	114.0<br>-143.0
-1.60<br>-1.42	170.0<br>-109.0

Table S3. [Ru(bpy)$_2$Cl(dmpzH)]OTf, **1c**

V(x) max. and min. (V)	Intensity (µA)
20 mV/s	
0.739<br>0.817	3.47<br>-15.3
-1.54<br>-1.60	20.3<br>10.9
50 mV/s	
0.739<br>0.817	8.26<br>-25.6
-1.55<br>-1.48	28.6<br>-0.04
100 mV/s	
0.739<br>0.817	17.7<br>-35.2
-1.56<br>-1.48	40.4<br>-11.0
200 mV/s	
0.736<br>0.824	30.0<br>-50.6
-1.57<br>-1.45	57.0<br>-23.3
500 mV/s	
0.719<br>0.834	58.0<br>-78.7
-1.58<br>-1.44	93.5<br>-43.1
1000 mV/s	
0.712<br>0.855	85.1<br>-111.0
-1.59<br>-1.45	134.0<br>-62.3

Table S4. $[Ru(bpy)_2(NH{=}C(Me)pz\text{-}\kappa^2N,N)](OTf)_2$, **3a**

V(x) max. and min. (V)	Intensity (μA)
20 mV/s	
1.16	3.5
1.24	-19.4
-1.44	20.8
-1.51	1.3
-1.62	37.2
-1.70	22.4
50 mV/s	
1.16	12.0
1.24	-28.4
-1.46	33.1
-1.38	-11.1
-1.67	52.3
-1.52	-7.2
100 mV/s	
1.15	22.0
1.25	-40.6
-1.47	47.9
-1.38	-27.9
-1.67	74.9
-1.52	-13.7
200 mV/s	
1.15	33.0
1.26	-59.2
-1.47	68.0
-1.37	-50.5
-1.68	106.0
-1.54	-22.6
500 mV/s	
1.14	56.8
1.27	-94.6
-1.49	112.0
-1.36	-87.1
-1.70	173.0
-1.55	-36.9
1000 mV/s	
1.12	78.5
1.29	-136.0
-1.51	159.0
-1.35	-128.0
-1.72	251.0
-1.54	-50.9

Table S5. [Ru(bpy)$_2$(NH=C(Me)IndzH-κ^{2}N,N)](OTf)$_2$, **3b**

V(x) max. and min. (V)	Intensity (μA)
20 mV/s	
1.15<br>1.23	7.4<br>-21.2
-1.45<br>-1.50	27.8<br>-0.4
-1.63<br>-1.71	43.1<br>19.8
50 mV/s	
1.15<br>1.23	18.0<br>-34.3
-1.46<br>-1.38	41.1<br>-17.6
-1.64<br>-1.69	63.7<br>18.4
100 mV/s	
1.15<br>1.23	30.2<br>-48.7
-1.46<br>-1.37	60.5<br>-39.8
-1.65<br>-1.50	93.1<br>-21.0
200 mV/s	
1.14<br>1.24	41.0<br>-70.9
-1.46<br>-1.37	83.0<br>-65.0
-1.66<br>-1.50	131.0<br>-32.0
500 mV/s	
1.14<br>1.26	68.1<br>-114.0
-1.48<br>-1.36	136.0<br>-112
-1.68<br>-1.55	215.0<br>-47.9
1000 mV/s	
1.12<br>1.28	90.0<br>-164.0
-1.50<br>-1.34	192.0<br>-162.0
-1.71<br>-1.54	310.0<br>-66.1

Table S6. $[Ru(bpy)_2(NH{=}C(Me)dmpzH\text{-}\kappa^2N,N)](OTf)_2$, **3c**

V(x) max. and min. (V)	Intensity (μA)
20 mV/s	
1.09<br>1.18	8.1<br>-14.6
-1.41<br>-1.42	17.0<br>-13.2
50 mV/s	
1.09<br>1.18	14.1<br>-22.3
-1.44<br>-1.47	25.4<br>-13.0
100 mV/s	
1.08<br>1.18	20.5<br>-33.0
-1.47<br>-1.50	39.0<br>-18.8
200 mV/s	
1.06<br>1.21	29.9<br>-52.3
-1.49<br>-1.49	54.0<br>-26.8
500 mV/s	
1.05<br>1.23	46.6<br>-75.4
-1.53<br>-1.48	91.8<br>-35.0
1000 mV/s	
1.03<br>1.26	62.9<br>-114.0
-1.55<br>-1.47	127.0<br>-36.4

Table S7. [Ru(bpy)$_2$(NH=C(Ph)pz-κ^{2}N,N)](OTf)$_2$, **4a**

V(x) max. and min. (V)	Intensity (μA)
20 mV/s	
1.17<br>1.28	11.1<br>-20.0
-1.43<br>-1.45	21.6<br>-9.5
-1.60<br>-1.65	50.3<br>25.4
50 mV/s	
1.17<br>1.29	20.0<br>-34.0
-1.44<br>-1.46	42.3<br>-15.9
-1.62<br>-1.67	80.0<br>21.3
100 mV/s	
1.16<br>1.31	30.0<br>-51.2
-1.45<br>-1.46	52.3<br>-28.5
-1.64<br>-1.67	101.2<br>20.7
200 mV/s	
1.15<br>1.35	39.2<br>-59.6
-1.45<br>-1.47	82.2<br>-39.7
-1.66<br>-1.68	104.2<br>10.2
500 mV/s	
1.11<br>1.38	68.3<br>-101.2
-1.47<br>-1.50	87.2<br>-49.0
-1.67<br>-1.69	132.9<br>-5.2
1000 mV/s	
1.10<br>1.42	80.7<br>-147.3
-1.48<br>-1.50	94.8<br>-64.6
-1.69<br>-1.71	155.0<br>-10.9

Table S8. $[Ru(bpy)_2(NH{=}C(Ph)Indz\text{-}\kappa^2N,N)](OTf)_2$, **4b**

V(x) max. and min. (V)	Intensity (μA)
20 mV/s	
1.21<br>1.27	2.0<br>-13.9
-1.43<br>-1.45	14.6<br>0.3
-1.60<br>-1.65	25.3<br>11.4
50 mV/s	
1.20<br>1.27	8.0<br>-21.3
-1.44<br>-1.46	21.0<br>-8.0
-1.62<br>-1.67	33.9<br>7.6
100 mV/s	
1.19<br>1.28	14.9<br>-30.8
-1.45<br>-1.46	29.7<br>-14.5
-1.63<br>-1.66	47.2<br>7.5
200 mV/s	
1.18<br>1.28	22.0<br>-44.5
-1.45<br>-1.46	40.0<br>-21.9
-1.64<br>-1.66	66.0<br>10.9
500 mV/s	
1.17<br>1.30	38.3<br>-74.1
-1.47<br>-1.49	67.2<br>-41.0
-1.67<br>-1.70	109.0<br>-33.9
1000 mV/s	
1.16<br>1.31	51.7<br>-104.0
-1.48<br>-1.50	94.8<br>-64.6
-1.69<br>-1.70	155.0<br>-42.9

Table S9. [Ru(bpy)$_2$(NH=C(Ph)dmpz-κ^{2}N,N)](OTf)$_2$, **4c**

V(x) max. and min. (V)	Intensity (μA)
20 mV/s	
1.14<br>1.21	2.3<br>-15.3
-1.44<br>-1.50	15.2<br>0.8
-1.59<br>-1.67	28.7<br>13.5
50 mV/s	
1.14<br>1.22	9.1<br>-22.9
-1.45<br>-1.37	21.8<br>-3.4
-1.64<br>-1.69	37.7<br>15.7
100 mV/s	
1.13<br>1.22	16.8<br>-33.0
-1.45<br>-1.50	30.3<br>-12.3
-1.64<br>-1.72	50.7<br>21.8
200 mV/s	
1.12<br>1.23	26.1<br>-48.8
-1.46<br>-1.50	41.0<br>-18.4
-1.67<br>-1.70	71.0<br>25.0
500 mV/s	
1.11<br>1.25	43.0<br>-78.3
-1.48<br>-1.48	66.9<br>-32.0
-1.70<br>-1.48	114.0<br>-26.0
1000 mV/s	
1.10<br>1.27	59.0<br>-114.0
-1.50<br>-1.36	94.9<br>-50.2
-1.72<br>-1.48	163.0<br>-35.0

Light system for performing the photocatalytic reactions

The photocatalytic system used for performing the photochemical reactions was a custom-made temperature-controlled system, in which the reaction mixture was kept at room temperature by passing coolant through the metallic system employing a recirculating chiller. The irradiation was achieved with a 5000 K single white LED located 1 cm beneath the base of the vial (Figure S25).

The emission spectrum of the light sources used for the photochemical reactions was recorded on an optical spectrometer *StellarNet model Blue-Wave UV-NB50.*

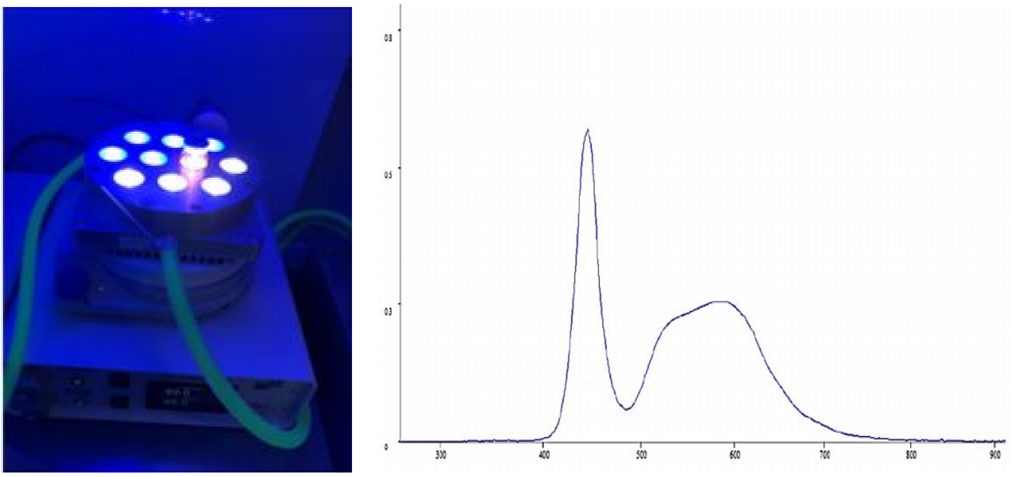

Figure S25. Custom-made temperature-controlled photocatalytic system (left). Emission spectrum of the 5000 K White LED (right). The picture has been taken by the authors of the manuscript.

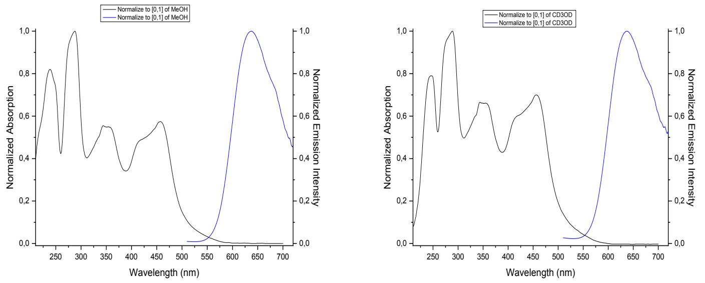

Figure S26. Normalized UV/vis absorption (black) and emission (blue, λ_{ex} = 420 nm) spectra of **3c** at 298 K, in deaerated MeOH (left) and CD_3OD (right) in optically dilute solutions.

Table S10. Quantum yields and lifetimes of **3c** in MeOH and CD_3OD.

**Comp**	**Solvent**	**Ø ×10^{-2}**	**τ / ns**
**3c**	MeOH	1.4	131
**3c**	CD_3OD	3.1	136

Table S11. Mechanistic tests using scavengers.

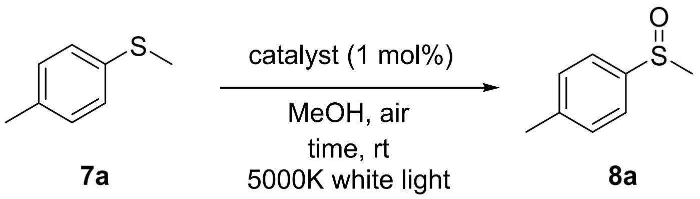

Entry	Additive (0.5 equiv)	Aim	Conversion (%)[a]		
			**3a**[b]	**3b**[c]	**3c**[d]
1	-		15	10	60
2	NaN_3	$^1O_2$ scavenger	0	0	0
3	1,4-dimethoxybenzene	R_2S^+ scavenger	20	4	53
4	benzoquinone	O_2^- scavenger	4	0	0

[a] Conversion determined by ^1H NMR analysis of the crude mixture. [b] Reaction stopped after 30 min. [c] Reaction stopped after 20 min. [d] Reaction stopped after 10 min.
